# CO I Barcoding Reveals New Clades and Radiation Patterns of Indo-Pacific Sponges of the Family Irciniidae (Demospongiae: Dictyoceratida)

**DOI:** 10.1371/journal.pone.0009950

**Published:** 2010-04-01

**Authors:** Judith Pöppe, Patricia Sutcliffe, John N. A. Hooper, Gert Wörheide, Dirk Erpenbeck

**Affiliations:** 1 Museum für Naturkunde, Department of Malacozoology, Leibniz Institute for Research on Evolution and Biodiversity at the Humboldt University Berlin, Berlin, Germany; 2 Biodiversity Program, Queensland Museum, South Brisbane, Australia; 3 Department of Earth- and Environmental Sciences, Palaeontology and Geobiology & GeoBio-CenterLMU, Ludwig-Maximilians-University Munich, Munich, Germany; University of Edinburgh, United Kingdom

## Abstract

**Background:**

DNA barcoding is a promising tool to facilitate a rapid and unambiguous identification of sponge species. Demosponges of the order Dictyoceratida are particularly challenging to identify, but are of ecological as well as biochemical importance.

**Methodology/Principal Findings:**

Here we apply DNA barcoding with the standard CO1-barcoding marker on selected Indo-Pacific specimens of two genera, *Ircinia* and *Psammocinia* of the family Irciniidae. We show that the CO1 marker identifies several species new to science, reveals separate radiation patterns of deep-sea *Ircinia* sponges and indicates dispersal patterns of *Psammocinia* species. However, some species cannot be unambiguously barcoded by solely this marker due to low evolutionary rates.

**Conclusions/Significance:**

We support previous suggestions for a combination of the standard CO1 fragment with an additional fragment for sponge DNA barcoding.

## Introduction

Sponges (Porifera Grant, 1836) are an important group of Metazoa in which species identification based on morphological characters is particularly difficult. However, as a group they are highly diverse, ecologically important as filter feeders and of commercial importance to the pharmaceutical and biomaterials industry as producers of highly potent secondary metabolites e.g. [1]. Most sponge taxa possess only a depauperate suite of complex characters. The basis of poriferan morphological systematics and species identification is based on the skeletal elements, their size, shape, arrangement and combination. Unfortunately, the evolution of skeletal traits is not fully understood. Furthermore, the diversity of these skeletal elements is frequently small, patterns in arrangement are hardly detectable, and environment-induced morphological variability makes their unambiguous interpretation difficult (see e.g. [2], [3]). This often results in homoplasies and erroneous classification [4]. Collectively, these factors make Porifera highly susceptible to cryptic speciation [5], and the actual species diversity and radiation may be under-estimated [6], [7], [8].

Such problematic species identification applies particularly to “keratose” sponges, which comprise taxa of the orders Dendroceratida and Dictyoceratida (Minchin, 1900). Keratose sponges lack a mineral skeleton, known from most other demosponge groups, but possess an organic skeleton made of spongin fibers instead (see [9], [10] for more details). Such an organic skeleton provides less morphological complexity than its mineral counterparts and makes this group a special challenge even for experienced taxonomists. Additionally, the long-term storage of (type) specimens in desiccating preservatives such as ethanol results in changes to specimen colour and tissue shrinking, which makes morphological comparison difficult. As keratose sponges also produce a particularly wide range of bioactive compounds of particular interest for the pharmaceutical industries [11], means of unambiguous (i.e. non-morphological) species identification have to be employed.

Among the dictyoceratid sponges, the family Irciniidae Gray, 1867 has autapomorphic features distinguishing this taxon from other demosponge families: its taxa possess fine collagen filaments in the mesohyl, which gives the sponges a rubber-like texture. Family Irciniidae currently consists of three genera with 111 described species [12] with an assumed worldwide distribution. The genus *Ircinia* currently comprises over 77 described species [12] and differs from *Sarcotragus* Schmidt, 1862 (11 known species) by the nature of the primary fibers. *Psammocinia* Lendenfeld, 1889 for which 23 species are currently described, is distinguished by a dermis armoured with a thick crustose layer of foreign debris. However, the classification of the species is more difficult, increasing the probability of the existence of cryptic species among the known specimens [13], [14], and their detection purely by means of morphology appears unlikely.

A potential solution is provided by DNA taxonomic approaches such as DNA barcoding. DNA barcoding was introduced as a method not only for the identification of known species but also for discovery of cryptic speciation by means of diagnostic DNA sequences [15], [16]. For sponges, concerted barcoding has been set up only recently (www.spongebarcoding.org, [17], [18]), and is performed initially under usage of the 5′ region of CO1, the standard barcoding fragment [15]. However, the suitability of this CO1 fragment for sponge species remains to be evaluated given the reduced substitution rates for mitochondria in Porifera and Cnidaria [19] (see also [20]), which could diminish the resolution power at species- or genus level.

In this study we follow two goals: First we aim to estimate whether the resolution power of this standard CO1 barcoding fragment may be sufficient for Irciniidae, or whether the reduced mitochondrial substitution rates may prevent any molecular separation below genus level. Second, we aim to assess radiation pattern and evidence of cryptic speciation in Irciniidae. For this purpose we DNA-barcoded an Irciniidae selection from Australia and from other regions of the Indo-Pacific, which is a hotspot for keratose sponge radiation.

## Results

**Figure 1:**
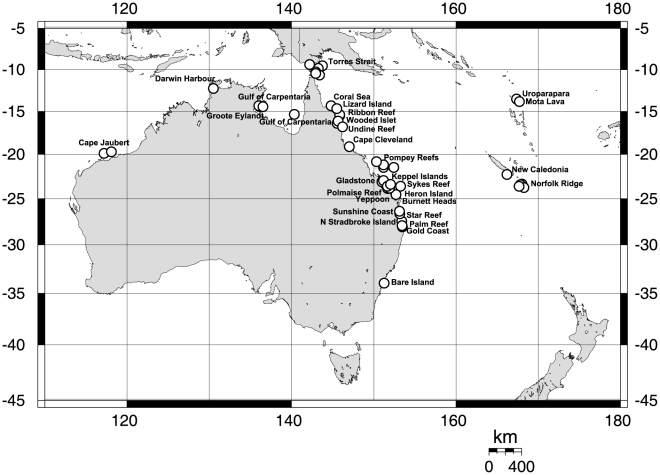
Map of the sampling locations. *Ircinia spiculosa* G311562 has been collected off Phuket, Thailand and is not indicated in the map for clarity reasons. See Table S1 for additional information.

The list of specimens, for which we succeeded in receiving amplifyable DNA and unambiguous sequences is given in Table S1. The specimens analyzed in this study originate from the Porifera collection of the Queensland Museum. The taxon set comprised specimens of the genera *Ircinia* and *Psammocinia*. Figure 1 provides an overview on their geographical distribution. The genus *Sarcotragus* was not included in analysis as its status is viewed as uncertain [14] and awaits revision. *Ircinia* and *Psammocinia* of different morphological groups have been selected, which were partially readily determined to species level, partially awaiting their new species description or final determination. In the following we will refer to these as “species” based on their significant morphological distinctness, which is frequently greater than the morphological differences observed in most well-established (i.e. biological) species (e.g. *Halichondria panicea* and *H. bowerbanki* [21]). In other studies these “species” have been referred to as Operational Taxononomic Units, or OTUs. See File S1 for details on the species.

**Figure 2:**
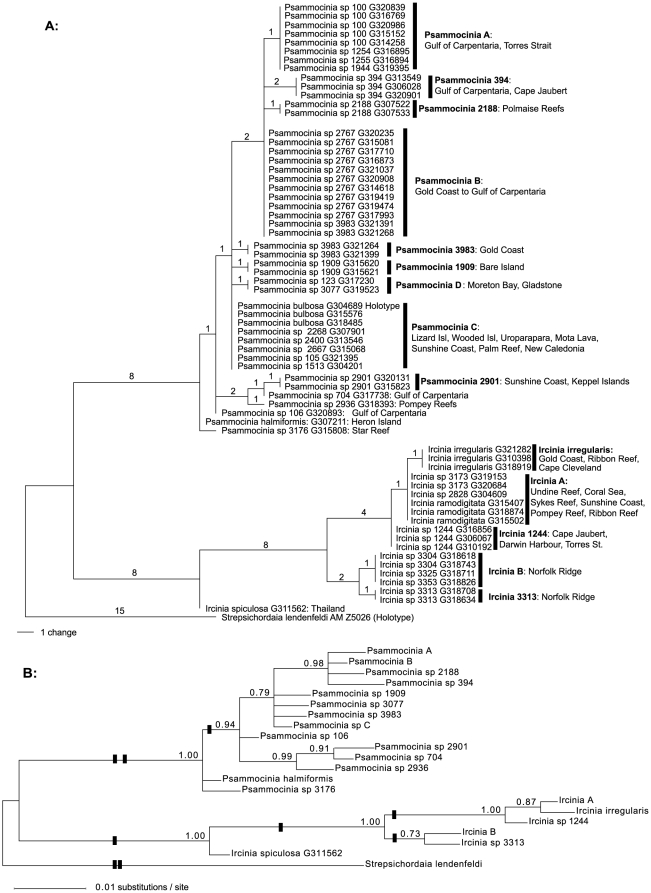
Phylogenetic reconstructions. A. Maximum-parsimony phylogram with the genetic differences of the specimens and the contents of the haplotypes. Haplotypes were named after their species, or in the case of several species per haplotype, with a letter. Species names are followed by their Queensland Museum species number, and the QM-collection number (Gxxxxxx). Numbers above the branches indicate total differences (in substitutions). B. Bayesian inference phylogram on the haplotype relationships. Haplotype names refer to figure A. The numbers above the branches are posterior probabilities. Black bars indicate amino acid changes.

The final dataset for the haplotype analysis consisted of 66 specimens and 519 characters of CO1. We differentiated a total of 6 different haplotypes of *Ircinia* and 14 haplotypes of *Psammocinia*. The phylograms in Figure 2 show haplotypes and phylogenetic relationships as reconstructed by parsimony- and bayesian methods. The phylogenetic trees display a clear distinction between the *Psammocinia* clade and the *Ircinia* clade. The *Ircinia* clade consists of six shallow water (<60 m) species with *I. spiculosa* from Thailand as a sister-group to the remaining *Ircinias*. The remaining five shallow water species are from Australia and comprise three haplotypes, one of which is shared by *I. ramodigitata* Burton, 1934 and species 3173 and 2828. There is no intraspecific variation in any of the species, while interspecific variation comprises 0–0.4% (p-distances, see also Table S2).

Figure 2 displays a distinctive split between the Australian shallow water sponges and four species collected from the seamounts of the Norfolk Ridge, south of New Caledonia (Figure 1). These deep-sea (180–470 m) specimens possess two distinct haplotypes, which differ by 2 basepairs (0.4%) and represent species likely to be new to science.

In total the genetic variation inside the *Ircinia* clade comprises not more than 2.7% (p-distance) difference between the species. Three substitutions resulted in non-synonymous codon changes and affected the protein sequence (see Figure 2B).

The 46 sequences of the genus *Psammocinia* resulted in 14 distinct haplotypes (Figure 2). Ten haplotypes could be assigned to distinct *Psammocinia* species that were supported by their morphological species groups, whereas four showed distinct morphological variation within their haplotypes. Only in one case it was found that four specimens of a supposedly morphologically distinctive *Psammocinia* species were split into two haplotypes (species 3983). Several haplotypes comprise geographically distinct locations (e.g. 1909, 2188). In total the genetic variation among *Psammocinia* species comprises 0–1.8% difference between the species. Only one substitution resulted in changes of the protein sequence within the *Psammocinia* clade (see Figure 2B).

## Discussion

Our results show that the standard barcoding marker cf. [15] is an important tool to detect cryptic speciation and to aid taxonomy. On the supra-specific level, the CO1 fragment provides clear separation between the target genera and facilitates the correct identification of genera, which is a problem in several other poriferan taxa such as the Haplosclerida, in which mitochondrial CO1 haplotypes fail to provide clear distinction between (morphologically) accepted genera or even families [22]. Here, *Psammocinia* and *Ircinia*, which morphologically differ only by the absence or presence of an armoured ectosome, are neatly divided into two clades. Even though the generic assignments of keratose taxa is problematic for many taxonomists and can only be achieved with histological sections and long experience, we have demonstrated that the standard CO1 barcoding fragment is suitable at this level, although more genera will need to undergo additional testing.

At the species level, the main target of DNA-barcoding projects, the standard CO1 fragment resolved several *Ircinia* and *Psammocinia* species from each other. The low genetic differences between these dictyoceratid haplotypes indicate the insufficient resolution power of CO1 for the species analysed in this data set. The interspecific variation of 2.7 and 1.8% for *Ircinia* and *Psammocinia* respectively, is clearly below the variation of 10–20% interspecific distances suggested for molecular distinction of species in other non-bilaterian Metazoa [23], [24]. A suitable barcoding gap [25], which distinguishes between the species and the next higher taxon, is therefore difficult to find. However, interspecific distances in a sufficient range have been found among other demosponge genera, such as *Scopalina* (Order Halichondrida), in which up to 22% sequence divergence (uncorrected) among OTUs has been detected [8] (see also genus *Tethya* (Order Hadromerida) [26]).

Our results suggest that the barcoding fragment may be too conserved to provide unambiguous barcodes for every demosponge species, because some haplotypes in our analysis are shared by several otherwise morphologically divergent species. For example, clade “*Psammocinia* C” contains *P. bulbosa* and 5 other so far unnamed species, which are clearly morphologically distinct from *P. bulbosa*, yet share the same CO1 haplotype.

On this basis, the CO1 haplotypes do not result in species-specific barcodes, because several *Ircinia* and *Psammocinia* species share the same haplotype. Therefore it is evident that the CO1 standard barcoding marker is not suitable as the only barcoding marker (at least not for the taxa investigated here). Where several species share a haplotype, additional markers should be used in combination with the standard CO1 fragment to provide better species-level resolution within the CO1 haplotype [18]. Suggestions for an alternative marker include ITS [27] (but see also [28]) or an additional 3′ region of the CO1 fragment [29] which has already been successfully tested in *Xestospongia* (Haplosclerida) [30].

A deep split between shallow and deep-sea species from the Norfolk Ridge seamounts was demonstrated here. In the literature, seamounts such as the Norfolk Ridge, are regarded as deep sea “islands” with unique biodiversity and restricted species ranges [31], and new bioactive compounds were detected from Norfolk Ridge *Ircinia* [32]. Our current data provides evidence for a radiation among Norfolk Ridge species since the four species included here form a monophyletic group with internal genetic differentiation. This pattern raises evidence for a single separation event from other *Ircinia*, although not much more can be deduced from the presently limited data set.

Furthermore, an extensive radiation of *Psammocinia* in Australian waters is indicated by our data. Currently there are three described species from Australia (*Psammocinia arenosa* (Lendenfeld, 1888) and *P. vesiculifera* (Poléjaeff, 1884) from New South Wales, and *P. halmiformis* (Lendenfeld, 1888) from Western Australia – none of which occur in tropical waters as do most of the species investigated here– and one from New Caledonia (*P. bulbosa* Bergquist, 1995). All other known *Psammocinia* species are from New Zealand, South Korea and Brazil. Therefore, most of the species investigated here undoubtedly represent new taxa. Clearly, the number of described *Psammocinia* species of Australian (and other) waters does not reflect the total biodiversity of this genus. *Psammocinia* sp. 3983 is the only species in our data set with two haplotypes and may be another example of cryptic speciation in sponges [6], [8], [33] considering the comparatively vast divergence (3 nucleotide substitutions) between both haplotypes.

In addition, some *Psammocinia* species appear to be geographically restricted. Several *Psammocinia* haplotypes represent specimens from a narrow geographical range (e.g. 1909, 2188 Figure 2), reflecting species-level differentiation. Other populations of the same species would have been indicated by the same haplotype, because intraspecific variation in demosponge CO1 is low. For example, nucleotide diversities among populations of *Crambe crambe* (Poecilosclerida) and *Astrosclera willeyana* (Agelasida) collected from locations several thousand kilometres apart were found as low as π = 0.00049 and π = 0.0006, respectively [34], [35]. This is in congruence with current views that geographic ranges of sponge species are frequently overestimated [36] and the number of distinct species is higher than expected [37], [38].

In conclusion, at the dawn of sponge barcoding our data indicate the great potential for DNA technologies to assist in resolving the taxonomy of sponges. From the examples of *Ircinia* and *Psammocinia* we demonstrate that barcoding facilitates rapid assessment of biodiversity, radiation patterns and the detection of cryptic speciation. However, the CO1 standard barcoding fragment should be used in combination with another DNA marker in order to achieve unambiguous taxonomic identification at species level.

## Materials and Methods

All specimens were collected by scuba, trawl, or dredge. A piece of about 3 mm^3^ was taken and DNA was extracted with the DNeasy Tissue kit by QIAGEN (Hilden, Germany) following the protocol for animal tissue. Among the specimens of the taxon set is the holotype of *Psammocinia bulbosa* (G304689) and *Strepsichordaia lendenfeldi* (Z5026, Thorectidae), the latter was used as the outgroup for phylogenetic reconstructions. The CO1 fragments were amplified using a twofold-degenerated version of the universal barcoding primers: dgLCO1490 (GGT CAA CAA ATC ATA AAG AYA TYG G) and dgHCO2198 (TAA ACT TCAG GGT GAC CAA ARA AYC A) [25] with an annealing temperature of 43°C. The PCR product was purified in a second step with silica based method described in Boyle & Lew, 1995. The sequencing reaction was performed with the BigDye-Terminator Mix v3.1 (ABI) following the manufacturers protocol. The template was sequenced on an ABI 3100 automated sequencer. The poriferan origin of the sequences was checked by a BLAST search [39] against the NCBI Genbank collection (http://www.ncbi.nlm.nih.gov/). Only sponge sequences were analyzed. Sequences were base-called, clipped and assembled by CodonCode Aligner v 2.0.4. MacClade v.4.06 [40] was used for the sequence management including the estimation of haplotype frequency in the data set. Sequences were aligned in Sea-View [41] using the Muscle [42] algorithm. Due to the protein coding nature of the sequence, the alignment has been unambiguous. Sequences are deposited in the European Molecular Biology Laboratory (EMBL) under accession numbers FN552810 - FN552875, together with the photographic documentation in the sponge barcoding database (www.spongebarcoding.org). Evolutionary distances and parsimonious tree reconstructions were performed with PAUP 4b10 [43] using heuristic searches in order to display haplotype diversity and relationships.

Haplotypes were phylogenetically reconstructed with Bayesian inference methods using MrBayes 3.12b [44] under the HKY+G model as suggested by Modeltest 3.7 [45]. Two runs with four Metropolis-coupled chains each were run until the standard deviation of split frequencies dropped below 0.01. Trees were burned in until the distribution of topology likelihoods reached the plateau phase. The map was drawn with MAKE_MAP (http://www.aquarius.ifm-geomar.de/make_map.html).

## Supporting Information

Table S1 Species list and collection details of the samples included in the data set.
(0.06 MB PDF)

Table S2 Pairwise distances of the haplotypes. Irc = Ircinia, Psam = Psammocinia, halm = halmiformis. Top right: p-distances, bottom left: total differences.
(0.02 MB PDF)

File S1 Morphological features of the yet undescribed species of the Psammocinia and Ircinia in the analysis.
(2.08 MB PDF)
