# Supplementary material for: CO I Barcoding Reveals New Clades and Radiation Patterns of Indo-Pacific Sponges of the Family Irciniidae (Demospongiae: Dictyoceratida)
Source: PLoS One. 2010 Apr 1;5(4):e9950. doi: 10.1371/journal.pone.0009950 (PMC2848591; doi:10.1371/journal.pone.0009950)
Supplement: Table S1 — Species list and collection details of the samples included in the data set. (0.06 MB PDF) [file pone.0009950.s001.pdf]

Species name	Sp. number	AccessionNo	Year	Location	Collector	Method	Latitude	Longitude	Depth
Ircinia irregularis		G310398	1988	Cape Cleveland/QLD/Australia			19° 6' 28.8" S	147° 2' 9.6" East	14m
Ircinia irregularis		G318919	2001	Ribbon Reef /QLD/Australia	Hooper, J N A, Cook, S D, Woerheide, G, Schlacher, M and Edson, D	SCUBA	15° 22' 51.6"	145° 50' 27.78" East	27m
Ircinia irregularis		G321282	2004	Southern Gold Coast/QLD/Australia	Crowther, A, Ekins, M, Carini, G and Sutcliffe, P	SCUBA	28° 6' 27.3" S	153° 28' 37.8" East	12m - 13.7m
Ircinia ramodigitata		G315407	1999	Sunshine Coast/Queensland/Australia	Cook, S. D., Kennedy, J. A. Adams, C. L., Woerheide, G. and Edson, D.	SCUBA	20° 49' 30''So	150° 20' 06''East	19m
Ircinia ramodigitata		G315502	1999	Pompey Reefs/QLD/Australia	Cook, S D, Kennedy, J A Adams, C L, Woerheide, G and Edson, D	SCUBA	21° 28' 41.7"	151° 11' 17.09" East	18m
Ircinia ramodigitata		G318874	2001	Ribbon Reef /QLD/Australia	Hooper, J N A, Cook, S D, Woerheide, G, Schlacher, M and Edson, D	SCUBA	15° 28' 48.9"	145° 50' 51.29" East	28m
Ircinia sp.	1244	G306067	1995	Cape Jaubert/WA/Australia	Cook, S D on CSIRO RV Southern Surveyor	Trawl	19° 41' 7" Sou	118° 6' 5" East	51m
Ircinia sp.	1244	G310192	1987	Darwin Harbour/NT/Australia			12° 15' 18" So	130° 28' 51.6" East	10m
Ircinia sp.	1244	G316856	2004	Torres Strait/QLD/Australia	CSIRO Torres Strait Map_GM_01_2004 RV Gwendoline May	Trawl	9° 22' 48" Sou	142° 12' 0" East	5m
Ircinia sp.	2828	G304609	1992	Sykes Reef/Queensland/ Australia	Pettit, G.R. et al., Cancer Research Institute	SCUBA	23° 26' 10'' So	152° 04' 24'' East	25m
Ircinia sp.	3173	G319153	2001	Undine Reef/QLD/Australia	Hooper, J N A, Cook, S D, Woerheide, G, Schlacher, M and Edson, D	SCUBA	16° 6' 33.24"	145° 38' 37.24" East	16m
Ircinia sp.	3173	G320684	2003	Coral Sea/QLD/Australia	Hooper, J N A, Cook, S D, Schlacher, M, Richer de Forges, M, Crowther, A and Bartlett, C	SCUBA	14° 18' 8.7" S	144° 48' 48.85" East	23m
Ircinia sp.	3304	G318618	2001	Norfolk Ridge Seamount/Pacific Ocean Seamounts and Reefs	Richer de Forges, B, ORSTOM	Trawl	23° 22' 26.94'	168° 2' 29.58" East	204m - 216m
Ircinia sp.	3304	G318743	2001	Norfolk Ridge Seamount/Pacific Ocean Seamounts and Reefs	Richer de Forges, B, ORSTOM	Dredge	23° 22' 34.8"	168° 2' 2.99" East	180m - 250m
Ircinia sp.	3313	G318634	2001	Norfolk Ridge Seamount/Pacific Ocean Seamounts and Reefs	Richer de Forges, B, ORSTOM	Trawl	23° 36' 38.34'	167° 41' 12.2" East	463m - 470m
Ircinia sp.	3313	G318708	2001	Norfolk Ridge Seamount/Pacific Ocean Seamounts and Reefs	Richer de Forges, B, ORSTOM	Dredge	23° 45' 11.28'	168° 16' 29.65" East	400m - 420m
Ircinia sp.	3325	G318711	2001	Norfolk Ridge Seamount/Pacific Ocean Seamounts and Reefs	Richer de Forges, B, ORSTOM	Dredge	23° 45' 11.28'	168° 16' 29.65" East	400m - 420m
Ircinia sp.	3353	G318826	2001	Norfolk Ridge Seamount/Pacific Ocean Seamounts and Reefs	Richer de Forges, B, ORSTOM	Dredge	23° 27' 1.8" S	167° 50' 2.4" East	276m - 350m
Ircinia spiculosa		G311562	1990	E. Of Phuket Is./Thailand			8° 3' 0" South	98° 18' 57.6" East	6m - 15m
Psammocinia bulbosa		G315576	1999	Uroparapara Island/Vanuatu	Kennedy, J A and Woerheide, G	SCUBA	13° 31' 29.22'	167° 20' 42.61" East	19.9m - 51m
Psammocinia bulbosa		G318485	1999	Mota Lava Island/Vanuatu	Menou, J L and Laboute, P	SCUBA	13° 48' 17" So	167° 42' 10" East	27m
Psammocinia bulbosa		G304689	1977	New Caledonia	Laboute, P., ORSTOM Noumea		22° 18' 00''So	166° 10' 59'' East	42.5m
Psammocinia halmiformis		G307211	1996	Heron Island	Hooper J.N.A. Cook S.D. Kennedy J.A. and Tomkins P.A.	SCUBA	23° 25' 05''So	152° 03' 00'' East	25m
Psammocinia sp.	100	G314258	1998	Gulf of carpentaria/NT/Australia	S Leys	Trawl	14° 20' 0" Sou	136° 1' 60" East	20.5m
Psammocinia sp.	100	G315152	1998	Gulf of carpentaria/NT/Australia	Wassenberg, T CSIRO Cleveland	Trawl	14° 25' 4" Sou	136° 31' 37" East	30.9m
Psammocinia sp.	100	G316769	2004	Torres Strait/QLD/Australia	CSIRO Torres Strait Map_GM_01_2004 RV Gwendoline May	Trawl	10° 39' 0" Sou	143° 24' 36" East	28.6m
Psammocinia sp.	100	G320839	2003	Gulf of Carpentaria/QLD/Australia	Bartlett, C and Cook, S "Southern Surveyor" 2380403 CSIRO "Effects of Trawling"	Sled - Benthic	15° 20' 2.22"	140° 19' 49.98" East	28m
Psammocinia sp.	100	G320986	2004	Torres Strait/QLD/Australia	CSIRO Torres Strait Map_GM_01_2004 RV Gwendoline May	Trawl	9° 31' 12" Sou	143° 45' 36" East	37.1m
Psammocinia sp.	105	G321395	2004	Palm Reef/QLD/Australia	Crowther, A, Ekins, M, Carini, G and Sutcliffe, P	SCUBA	28° 6' 33.12"	153° 28' 30.96" East	10m - 24m
Psammocinia sp.	106	G320893	2003	Gulf of Carpentaria/QLD/Australia	Bartlett, C and Cook, S "Southern Surveyor" 2380403 CSIRO "Effects of Trawling"	Trawl	15° 20' 2.22"	140° 19' 49.98" East	28m
Psammocinia sp.	123	G317230	2000	N Stradbroke I/QLD/Australia	Hooper, J N A, Cook, S D, Kennedy, J A, List-Armitage, S, Edson, D and Woerheide, G	Hand Collectio	27° 28' 6" Sou	153° 24' 4" East	
Psammocinia sp.	394	G306028	1995	Cape Jaubert/WA/Australia	Cook, S D on CSIRO RV Southern Surveyor	Trawl	19° 54' 9" Sou	117° 12' 1" East	58m
Psammocinia sp.	394	G313549	1997	South of Groote Eylandt/NT/Australia	Cook, S D on CSIRO RV Southern Surveyor	Dredge	14° 27' 10.8"	136° 14' 16.82" East	22.5m
Psammocinia sp.	394	G320901	2003	Gulf of Carpentaria/Queensland/Australia	Bartlett, C. and Cook, S. "Southern Surveyor" 2380403 CSIRO "Effects of Trawling"	Trawl	15° 20' 02''No	140° 19' 50''East	28m
Psammocinia sp.	704	G317738	2001	Gulf of Carpentaria/Queensland/Australia	Hooper, Cook, Kennedy, Woerheide, Edson	SCUBA	21° 30' 00'' So	152° 26' 06'' East	30m
Psammocinia sp.	1254	G316895	2004	Torres Strait/QLD/Australia	CSIRO Torres Strait Map_GM_01_2004 RV Gwendoline May	Trawl	10° 27' 0" Sou	142° 55' 12" East	19m
Psammocinia sp.	1255	G316894	2004	Torres Strait/QLD/Australia	CSIRO Torres Strait Map_GM_01_2004 RV Gwendoline May	Trawl	10° 27' 0" Sou	142° 55' 12" East	19m
Psammocinia sp.	1513	G304201	1994	Lizard I/QLD/Australia	Hooper, J N A, Hobbs, L J, Kennedy, J and Cook, S D	SCUBA	14° 39' 0" Sou	145° 27' 0" East	18m
Psammocinia sp.	1909	G315620	1999	Bare Island/NSW/Australia	Taylor, M University of New South Wales	SCUBA	33° 59' 38" So	151° 13' 60" East	7m
Psammocinia sp.	1909	G315621	1999	Bare Island/NSW/Australia	Taylor, M University of New South Wales	SCUBA	33° 59' 38" So	151° 13' 60" East	7m
Psammocinia sp.	1944	G319395	2002	Cape Grafton/QLD/Australia	Hooper, J N A, Schlacher, M, Woerheide, G and Carroll, A et al	SCUBA	16° 48' 33.66'	146° 12' 46.79" East	32m
Psammocinia sp.	2188	G307522	1996	N. side Polmaise Reef/QLD/Australia	Hooper, J N A, Cook, S D, Kennedy, J A and Tomkins, P A	SCUBA	23° 33' 3" Sou	151° 39' 9" East	12m
Psammocinia sp.	2188	G307533	1996	N. side Polmaise Reef/QLD/Australia	Hooper, J N A, Cook, S D, Kennedy, J A and Tomkins, P A	SCUBA	23° 33' 3" Sou	151° 39' 9" East	12m
Psammocinia sp.	2268	G307901	1997	Wooded Islet/QLD/Australia	Hooper, J N A, Cook, S D, Kennedy, J A, Tomkins, P A and List-Armitage, S	SCUBA	16° 23' 4" Sou	145° 33' 8" East	
Psammocinia sp.	2400	G313546	1997	South of Groote Eylandt/NT/Australia	Cook, S D on CSIRO RV Southern Surveyor	Dredge	14° 27' 10.8"	136° 14' 16.82" East	22.5m
Psammocinia sp.	2667	G315068	1998	Sunshine Coast/QLD/Australia	Hooper, J N A, Cook, S D, Kennedy, J A and Woerheide, G	SCUBA	26° 39' 11.4"	153° 10' 59.78" East	19m
Psammocinia sp.	2767	G314618	1999	Gold Coast/QLD/Australia	Cook, S D, Kennedy, J A, Adams, C L and Woerheide, G	SCUBA	27° 57' 1" Sou	153° 26' 58.78" East	26m
Psammocinia sp.	2767	G315081	1998	Sunshine Coast/QLD/Australia	Hooper, J N A, Cook, S D, Kennedy, J A and Woerheide, G	SCUBA	26° 39' 11.4"	153° 10' 59.78" East	19m
Psammocinia sp.	2767	G316873	2004	Torres Strait/QLD/Australia	CSIRO Torres Strait Map_GM_01_2004 RV Gwendoline May	Trawl	10° 15' 0" Sou	142° 55' 48" East	22.1m
Psammocinia sp.	2767	G317710	2001	Swain Reefs/QLD/Australia	Hooper, J N A, Cook, S D, Kennedy, J A, Edson, D and Woerheide, G	SCUBA	21° 29' 53.46'	152° 24' 52.05" East	15m
Psammocinia sp.	2767	G317993		NE of Yeppoon/QLD/Australia	Queensland Department of Primary Industries Fisheries, Deception Bay (QDPI)	Trawl	22° 58' 26.4"	151° 10' 59.4" East	35m
Psammocinia sp.	2767	G319419		East of Gladstone/QLD/Australia	Queensland Department of Primary Industries Fisheries, Deception Bay (QDPI)	Trawl	23° 51' 35.4"	151° 41' 23.4" East	30m
Psammocinia sp.	2767	G319474		NE of Burnett Heads/QLD/Australia	Queensland Department of Primary Industries Fisheries, Deception Bay (QDPI)	Trawl	24° 32' 37.2"	152° 41' 28.79" East	28m
Psammocinia sp.	2767	G320235	2002	QDPI Scallop Survey/QLD/Australia	Queensland Department of Primary Industries Fisheries, Deception Bay (QDPI)	Trawl	23° 27' 4.42"	151° 28' 22.06" East	30m
Psammocinia sp.	2767	G320908	2003	Gulf of Carpentaria/QLD/Australia	Bartlett, C and Cook, S "Southern Surveyor" 2380403 CSIRO "Effects of Trawling"	Trawl	15° 20' 2.22"	140° 19' 49.98" East	28m
Psammocinia sp.	2767	G321037	2004	Torres Strait/QLD/Australia	CSIRO Torres Strait Map_GM_01_2004 RV Gwendoline May	Trawl	9° 50' 24" Sou	143° 14' 24" East	25.4m
Psammocinia sp.	2901	G315823	1999	Sunshine Coast/QLD/Australia	Cook, S D, Kennedy, J A, List-Armitage, S E, Adams, C L and Woerheide, G	SCUBA	26° 22' 32" So	153° 7' 33" East	29m
Psammocinia sp.	2901	G320131	2002	Keppel Islands/QLD/Australia	Hooper, J N A, Cook, S D, List-Armitage, S, Richer de Forges, M, Crowther, A and Ireland, H	SCUBA	23° 7' 5" Sout	150° 59' 27" East	17.4m
Psammocinia sp.	2936	G318393	2000	Pompey Reefs/QLD/Australia	Cook, S D, Kennedy, J A, Woerheide, G and Delaney, W	SCUBA	21° 12' 45" So	151° 11' 43" East	21.8m
Psammocinia sp.	3077	G319523		NE of Gladstone/QLD/Australia	Queensland Department of Primary Industries Fisheries, Deception Bay (QDPI)	Trawl	23° 36' 57" So	151° 38' 45.59" East	36m
Psammocinia sp.	3176	G315808	1999	Star Reef/Queensland/Australia	Cook, S. D., Kennedy, J. A., List-Armitage, S. E., Adams, C. L. and Woerheide, G.	SCUBA	26° 22' 12''So	153° 07' 30''East	29m
Psammocinia sp.	3983	G321264	2004	Southern Gold Coast/QLD/Australia	Crowther, A, Ekins, M, Carini, G and Sutcliffe, P	SCUBA	28° 6' 27.3" S	153° 28' 37.8" East	12.5m
Psammocinia sp.	3983	G321268	2004	Southern Gold Coast/QLD/Australia	Crowther, A, Ekins, M, Carini, G and Sutcliffe, P	SCUBA	28° 6' 27.3" S	153° 28' 37.8" East	12.5m
Psammocinia sp.	3983	G321391	2004	Palm Reef/QLD/Australia	Crowther, A, Ekins, M, Carini, G and Sutcliffe, P	SCUBA	28° 6' 33.12"	153° 28' 30.96" East	10m - 24m
Psammocinia sp.	3983	G321399	2004	Palm Reef/QLD/Australia	Crowther, A, Ekins, M, Carini, G and Sutcliffe, P	SCUBA	28° 6' 33.12"	153° 28' 30.96" East	10m - 24m
Strepsichordais lendenfeldi		AM Z5026		Daves Reef, Great Barrier Reef, QLD, Australia					
