# Supplementary material for: CO I Barcoding Reveals New Clades and Radiation Patterns of Indo-Pacific Sponges of the Family Irciniidae (Demospongiae: Dictyoceratida)
Source: PLoS One. 2010 Apr 1;5(4):e9950. doi: 10.1371/journal.pone.0009950 (PMC2848591; doi:10.1371/journal.pone.0009950)
Supplement: Table S2 — Pairwise distances of the haplotypes. Irc = Ircinia, Psam = Psammocinia, halm = halmiformis. Top right: p-distances, bottom left: total differences. (0.02 MB PDF) [file pone.0009950.s002.pdf]

	Taxon	1	2	3	4	5	6	7	8	9	10	11	12	13	14	15	16	17	18	19	20
1	**Irc A**	-	0.015	0.002	0.002	0.015	0.025	0.054	0.052	0.056	0.058	0.056	0.054	0.056	0.058	0.054	0.058	0.056	0.058	0.058	0.056
2	**Irc B**	8	-	0.013	0.017	0.004	0.019	0.048	0.046	0.05	0.052	0.05	0.048	0.05	0.052	0.048	0.052	0.046	0.052	0.052	0.05
3	**Irc sp 1244**	1	7	-	0.004	0.013	0.023	0.052	0.05	0.054	0.056	0.054	0.052	0.054	0.056	0.052	0.056	0.054	0.056	0.056	0.054
4	**Irc irregularis**	1	9	2	-	0.017	0.027	0.052	0.05	0.054	0.056	0.054	0.052	0.054	0.056	0.052	0.056	0.054	0.056	0.056	0.054
5	**Irc sp 3313**	8	2	7	9	-	0.019	0.048	0.046	0.05	0.052	0.05	0.048	0.05	0.052	0.048	0.052	0.046	0.052	0.052	0.05
6	**Irc spiculosa**	13	10	12	14	10	-	0.033	0.031	0.031	0.037	0.033	0.033	0.033	0.035	0.033	0.033	0.035	0.037	0.035	0.035
7	**Psam A**	28	25	27	27	25	17	-	0.002	0.01	0.008	0.008	0.004	0.015	0.014	0.008	0.012	0.006	0.008	0.013	0.006
8	**Psam B**	27	24	26	26	24	16	1	-	0.008	0.006	0.006	0.002	0.013	0.012	0.006	0.01	0.004	0.006	0.012	0.004
9	**Psam halmif**	29	26	28	28	26	16	5	4	-	0.006	0.002	0.01	0.01	0.008	0.006	0.002	0.012	0.006	0.008	0.004
10	**Psam sp 1909**	30	27	29	29	27	19	4	3	3	-	0.004	0.008	0.012	0.01	0.004	0.008	0.01	0.002	0.01	0.002
11	**Psam sp 106**	29	26	28	28	26	17	4	3	1	2	-	0.008	0.008	0.006	0.004	0.004	0.01	0.004	0.006	0.002
12	**Psam sp 2188**	28	25	27	27	25	17	2	1	5	4	4	-	0.015	0.014	0.008	0.012	0.006	0.008	0.013	0.006
13	**Psam sp 2901**	29	26	28	28	26	17	8	7	5	6	4	8	-	0.006	0.012	0.012	0.017	0.012	0.002	0.01
14	**Psam sp 2936**	30	27	29	29	27	18	7	6	4	5	3	7	3	-	0.01	0.01	0.016	0.01	0.004	0.008
15	**Psam sp D**	28	25	27	27	25	17	4	3	3	2	2	4	6	5	-	0.008	0.01	0.004	0.01	0.002
16	**Psam sp 3176**	30	27	29	29	27	17	6	5	1	4	2	6	6	5	4	-	0.013	0.008	0.01	0.006
17	**Psam sp 394**	29	24	28	28	24	18	3	2	6	5	5	3	9	8	5	7	-	0.01	0.015	0.008
18	**Psam sp 3983**	30	27	29	29	27	19	4	3	3	1	2	4	6	5	2	4	5	-	0.01	0.002
19	**Psam sp 704**	30	27	29	29	27	18	7	6	4	5	3	7	1	2	5	5	8	5	-	0.008
20	**Psam sp C**	29	26	28	28	26	18	3	2	2	1	1	3	5	4	1	3	4	1	4	-
