# Supplementary material for: CO I Barcoding Reveals New Clades and Radiation Patterns of Indo-Pacific Sponges of the Family Irciniidae (Demospongiae: Dictyoceratida)
Source: PLoS One. 2010 Apr 1;5(4):e9950. doi: 10.1371/journal.pone.0009950 (PMC2848591; doi:10.1371/journal.pone.0009950)
Supplement: File S1 — Morphological features of the yet undescribed species of the Psammocinia and Ircinia in the analysis. (2.08 MB PDF) [file pone.0009950.s003.pdf]

Ircinia sp1244

Material examined
QMG306067: Cape Jaubert/WA/Australia, 19° 41' 7" South, 118° 6' 5" East, 51m, 1995, Cook, S D on CSIRO RV Southern Surveyor, Trawl. QMG310192: Darwin Harbour/NT/Australia, 12° 15' 18" South, 130° 28' 51.6" East, 10m, 1987. QMG316856: Torres Strait/QLD/Australia, 9° 22' 48" South, 142° 12' 0" East, 5m, CSIRO Torres Strait Map_GM_01_2004 RV Gwendoline May, Trawl.

Description
Shape Subspherical, massive sponge.
Colour Grey, brown or olive to purple in situ. Beige to dark grey in ethanol. The ethanol is turned a clear yellow colour.
Oscules Large sieve plates clustered randomly on upper surface of sponge.
Texture and Surface characteristics Internally cavernous, resembling *Hyattella intestinalis.* The surface is skin like and smooth to touch with conules covering the entire surface at regular intervals. Areas between conules can take on a *Dysidea* like appearance as a result of the internal fibres being visible through the thin ectosomal layer. Very difficult to tear.
*Ectosome F*oreign spicules scattered regularly but sparsely in the membranous ectosomal layer.
Choanosome Cavernous, wide meshed reticulation of primary fibres. Tangled secondary fibres. All uncored. Collagenous filaments densely fill the mesohyl.

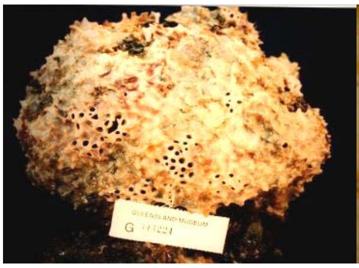
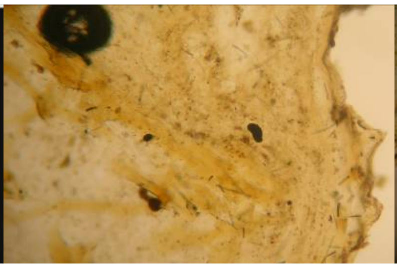
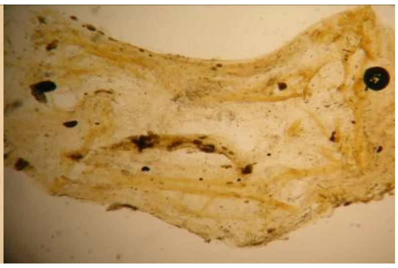

G303224 G303224 G303224

Ircinia sp3304

Material examined
QMG318618: Norfolk Ridge Seamount/Pacific Ocean Seamounts and Reefs, 23° 22' 26.94" South, 168° 2' 29.58" East, 204m - 216m, 2001, Richer de Forges, B, ORSTOM, Trawl. QMG318743: Norfolk Ridge Seamount/Pacific Ocean Seamounts and Reefs, 23° 22' 34.8" South, 168° 2' 2.99" East, 180m - 250m, 2001, Richer de Forges, B, ORSTOM, Dredge.

Description
Shape Pedunculate sponge which is firm and retains upright position. Basal attachment is slightly enlarged but not extensive. extending approx 2-3mm from the surface at random intervals.
Colour Purple colour in ethanol.
Oscules Bulbous terminally with protruding tubular oscules
Texture and Surface characteristics Surface is smooth and unornamented.
Ectosome The ectosome displays a slightly darkened layer of tissue but is unarmoured.
Choanosome The choanosome contains evenly reticulated primary and secondary fibres. Primary fibres are evenly cored and extend to the ectosome but do not protrude through the surface. Secondary tracts are clear and collagenous filaments are spread throughout the light mesohyl. Some specimens include echinating acanthostyles and poecilosclerid microscleres at the surface, however, no encrusting sponge can be seen. There is a photograph included of this, as it has also been observed in other deep sea sponges from the Norfolk Ridge and should not be ignored or confused with an encrusting ectosome of *Psammocinia*.

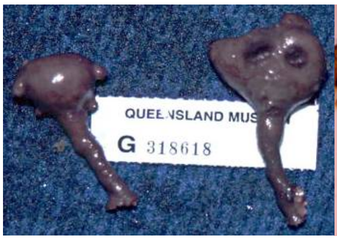

G318618

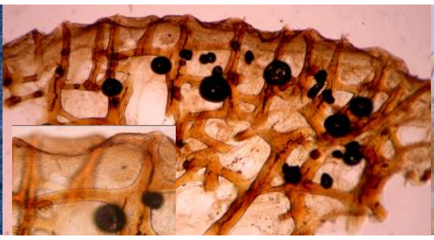
G318743

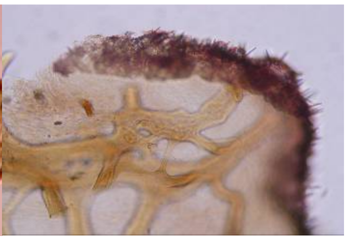
G318618

Ircinia sp3313

Material examined
QMG318634: Norfolk Ridge Seamount/Pacific Ocean Seamounts and Reefs, 23° 36' 38.34" South, 167° 41' 12.2" East, 463m - 470m, 2001, Richer de Forges, B, ORSTOM, Trawl. QMG318708 : Norfolk Ridge Seamount/Pacific Ocean Seamounts and Reefs, 23° 45' 11.28" South, 168° 16' 29.65" East, 400m - 420m, 2001, Richer de Forges, B, ORSTOM, Dredge.

Description
Shape Massive to globular shaped sponge.
Colour Light brown in ethanol
Oscules Oscules are not apparent.
Texture and Surface characteristics This sponge has a slightly compressible texture which is difficult to tear and a parchment like surface. Macroscopically the surface is very folded but in a semi regular way. Microscopically the surface is conulose and fibres can be seen at the tip of the conules.
Ectosome The surface is slightly darkened, similar to *Ircinia* sp3304, and can also contain foreign spicule inclusions in patches at the ectosome which are quite distinctive, but not to be confused with *Psammocinia*.
Choanosome The skeleton displays an irregular, sparse meshwork of primary and secondary fibres. Primary fibres are only lightly and sparsely cored, secondary fibres are clear. Collagenous filaments can be seen throughout the consistent, moderate to dense mesohyl.

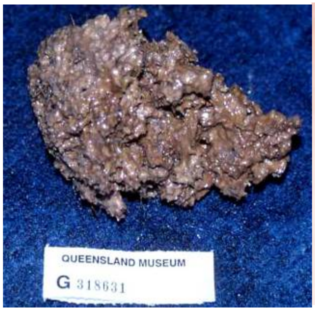

G318631

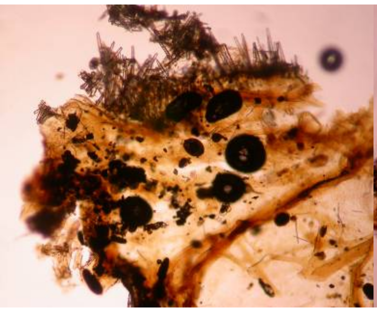

G318634

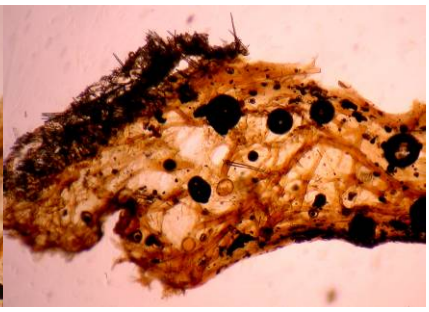

G318634

Ircinia sp3325

Material examined
QMG318711: Norfolk Ridge Seamount/Pacific Ocean Seamounts and Reefs, 23° 45' 11.28" South, 168° 16' 29.65" East, 400m - 420m, 2001, Richer de Forges, B, ORSTOM, Dredge.

Description
Shape Massive, digitate sponge
Colour Light purple colour in ethanol. The sponge also turns the ethanol brown
Oscules Small oscules are situated at the apex of the digitate projections.
Texture and Surface characteristics Compressible and conulose.
Ectosome A thin layer of collagen is slightly darkened at the ectosome. In patches, acanthostyles and microscleres form an echinating layer at the crust, similar to sp2204 and sp3313.
Choanosome Large, laminated fibres form a reticulation in the choanosome. Primary fibres are consistently cored, secondary fibres are clear. Collagenous filaments are dispersed throughout the mesohyl.

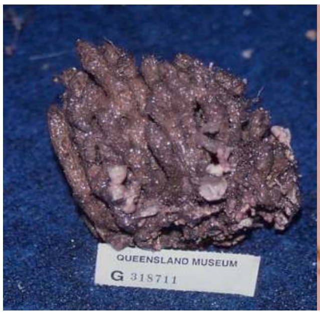

G318711

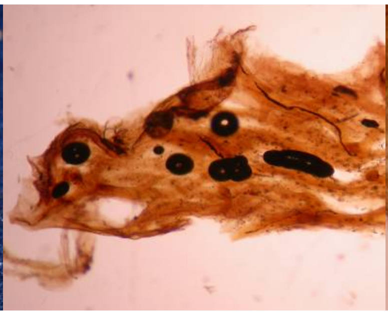
G318711

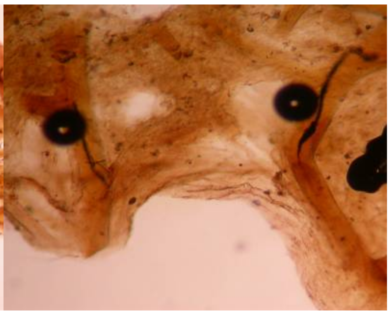
G318711

Ircinia sp3353

Material examined
QMG318826: Norfolk Ridge Seamount/Pacific Ocean Seamounts and Reefs, 23° 27' 1.8" South, 167° 50' 2.4" East, 276m - 350m, 2001,Richer de Forges, B, ORSTOM, Dredge.

Description
Shape Stalked, club shaped sponge.
Colour
Oscules One main apical oscule. Smaller oscules protrude from the surface to approx. 3mm and are all arranged to be opening towards the base of the sponge. These protrusions regularly and consistently cover the surface of the sponge.
Texture and Surface characteristics The sponge is firm and the surface of the sponge is similar to plastic and slightly transparent.
Ectosome The ectosome is darkened with a small amount of debris. A dense, consistent layer of collagen supports the ecotosome.
Choanosome Primary and secondary fibres large and homogenous, forming a reticulated pattern. Primary fibres are cored consistently, but lightly. Mesohyl and collagenous filaments light but consistent throughout choanosome.

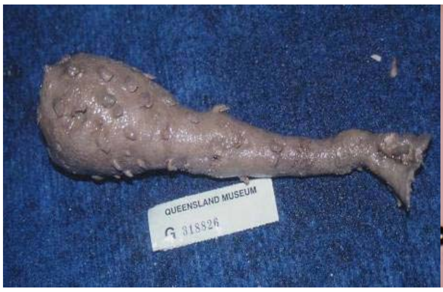

G318826

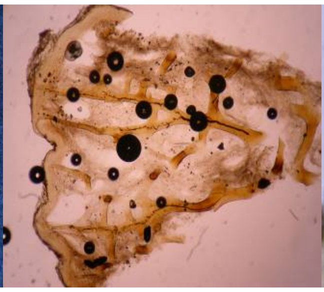

G318826

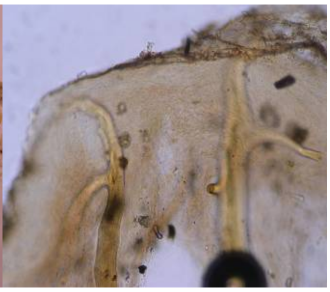

G318826

Ircinia cf spiculosa Hentschel, 1912

Material examined
QMG311562: E. Of Phuket Is./Thailand, 8° 3' 0" South, 98° 18' 57.6" East, 6m - 15m, 1990.

Description
Shape Massive, cavernous sponge.
Colour Cream in ethanol internally and externally and leaves the ethanol almost clear. Thin but prominent fibres are an orange colour in contrast to the cream colour of the main tissue.
Oscules Not apparent.
Texture and Surface characteristics The structure of this sponge is very characteristic as the body of the sponge is constructed of thick folds of tissue. Each of these folds tapers apically to very thin, tapering fistules which are highly conulose. Heavily cored primary fibres are a yellow colouration and are easily visible inside the thin fistules. The main body of the sponge is also conulose.
Ectosome Moderately thick crust comprised of foreign spicules which continue down the primary tracts which extend right up into the tips of the conules.
Choanosome Thick primary fibres cored predominantly with foreign spicules but also containing small sand grains. Extremely dense collagenous filaments creating a loosely reticulated pattern, obscured by foreign inclusions.

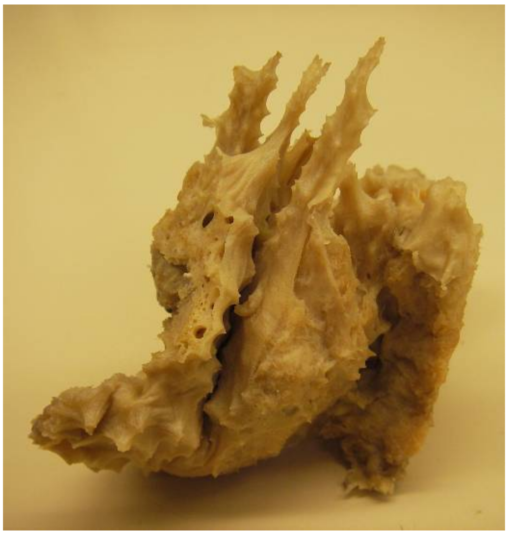

QMG311562

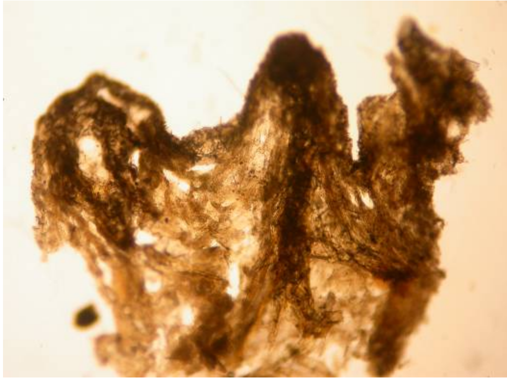

QMG311562

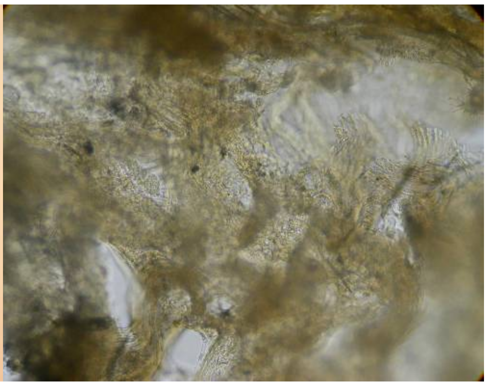

QMG311562

Psammocinia bulbosa Bergquist, 1995

Material examined
QMG304689 (Holotype): New Caledonia, 22° 18' 00"South, 166° 10' 59" East, 42.5m, 1977. Laboute, P., ORSTOM Noumea. QMG315576: Uroparapara Island/Vanuatu, 13° 31' 29.22" South, 167° 20' 42.61" East, 19.9m - 51m, 1999, Kennedy, J A and Woerheide, G, SCUBA. QMG318485: Mota Lava Island/Vanuatu, 13° 48' 17" South, 167° 42' 10" East, 27m, 1999, Menou, J L and Laboute, P, SCUBA.

Description
Shape. Sponge found spreading across and attached to the substrate, with bulbous sections rising up to tapering fistules.
Colour Grey to white colour in situ, turning predominantly white in ethanol.
Oscules Oscules are both flush with the surface and apical to the tapering fistules.
Texture and Surface characteristics Firm sponge which is slightly compressible with a dry texture due to the thick sandy cortex. The surface is regularly conulose, however the conules are low and rounded rather than pointed.
Ectosome Thick, even ectosomal crust of sand up to 1mm thick.
Choanosome Wide primary fibres are sparse, heavily cored and fasciculate. Secondary fibres can be either cored or clear and are irregularly arranged. Collagenous filaments are densely packed throughout the light collagenous mesohyl.

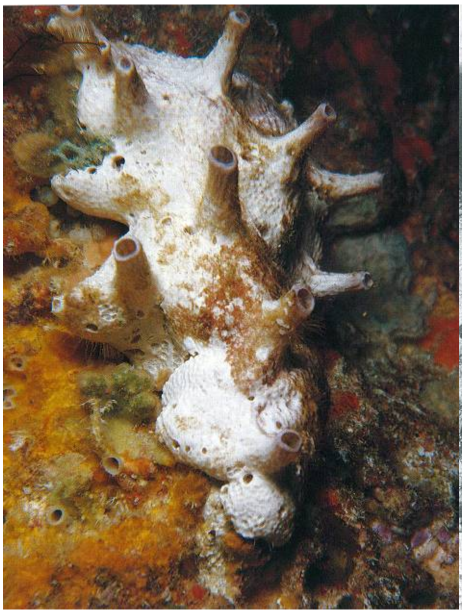

G304689

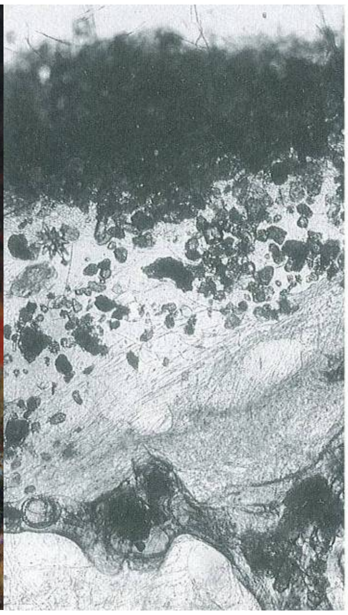

G304689

Psammocinia halmiformis (Lendenfeld, 1888)

Material examined
QMG307211: Heron Island, 23° 25' 05" South, 152° 03' 00" East, 25m, 1996, Hooper J.N.A. Cook S.D. Kennedy J.A. and Tomkins P.A., SCUBA.

Description
Shape Massive, ridge-like, flabellate sponge.
Colour Dark and light grey exterior in situ. Light tan-brown interior on deck. Light grey to white in ethanol.
Oscules Numerous, small, scattered oscules on the inner surface of the sponge.
Texture and Surface characteristics Firm, not very compressible sponge with no mucous. The surface is finely conulose on both the internal and external surfaces. The internal surface is otherwise relatively smooth and the external surface arranged in folds.
Ectosome Moderately thick, regular crust of sand.
Choanosome Semi regular reticulation of primary and secondary fibres. Primary fibres are cored with sand, extending into secondary fibres which are mostly clear. All fibres with distinctive light pith and concentric lamination. Mesohyl mostly clear, heavy with collagen filaments.

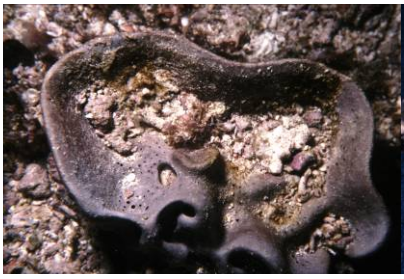

G307211

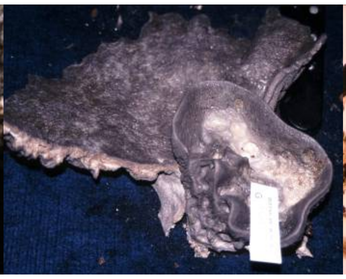

G307211

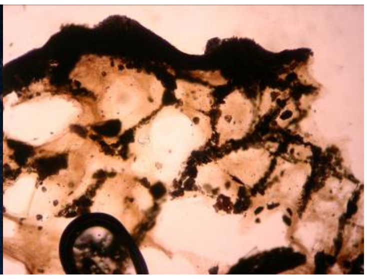

G307211

Psammocinia sp100

Material examined
QMG314258: Gulf of Carpentaria/NT/Australia, 14° 20' 0" South, 136° 1' 60" East, 20.5m, 1998, S Leys, Trawl. QMG315152: Gulf of Carpentaria/NT/Australia, 14° 25' 4" South, 136° 31' 37" East, 30.9m, 1998, Wassenberg, T CSIRO Cleveland, Trawl. QMG316769: Torres Strait/QLD/Australia, 10° 39' 0" South, 143° 24' 36" East, 28.6m, 2004 CSIRO Torres Strait Map_GM_01_2004 RV Gwendoline May, Trawl. QMG320839: Gulf of Carpentaria/QLD/Australia, 15° 20' 2.22" South, 140° 19' 49.98" East, 28m, 2003, Bartlett, C and Cook, S "Southern Surveyor" 2380403 CSIRO "Effects of Trawling", Sled – Benthic. QMG320986: Torres Strait/QLD/Australia, 9° 31' 12" South, 143° 45' 36" East, 37.1m, 2004, CSIRO Torres Strait Map_GM_01_2004 RV Gwendoline May, Trawl.

Description
Shape Massive sponge with folded surface.
Colour Beige to grey in ethanol.
Oscules Not apparent.
Texture and Surface characteristics Soft and flaccid sponge which feels gritty internally due to the foreign material coring the large fibres. This reticulation can be observed easily in the cut specimen. Large conules cover the surface, ranging from evenly distributed to large sweeping conules in some specimens. They all have distinctive ends, whether it be at a point, or more of a ridge, they all display quite pointed, conulose ends. See photograph.
Ectosome Foreign spicules form a distinct crust at the ectosome. These spicules are arranged in a very even, rounded, reticulated pattern.
Choanosome Reticulation of primary and secondary cored fibres. Patchy mesohyl.

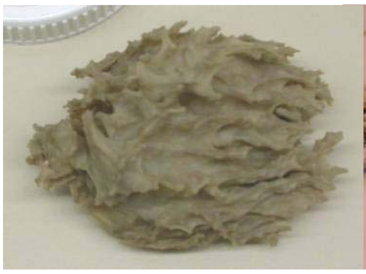

G320839

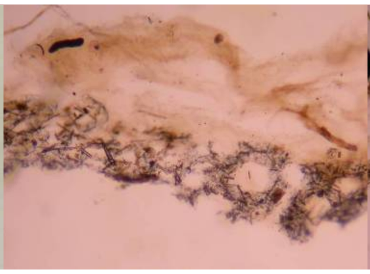

G314258

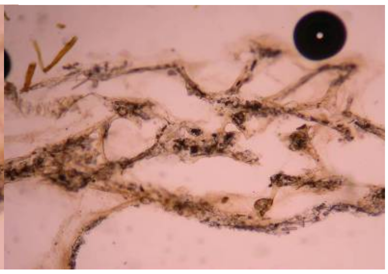

G314258

Psammocinia sp105

Material examined
QMG321395: Palm Reef/QLD/Australia, 28° 6' 33.12" South, 153° 28' 30.96" East, 10m - 24m, Crowther, A, Ekins, M, Carini, G and Sutcliffe, P, 2004, SCUBA.

Description
Shape Thickly encrusting sponge anostomosing over and incorporating the substrate, raised to a height of 2-3cm.
Colour Cream to grey colour in ethanol..
Oscules Oscules visible apical to the raised ridges.
Texture and Surface characteristics Compressible sponge which retains shape well. Conulose surface is partially opaque, giving a slightly mottled appearance due to the cavernous organisation within the body of the sponge.
Ectosome. Extremely fine crust of small sand grains forms a thin ectosomal layer.
Choanosome. Very large sand grains are found throughout the light mesohyl.

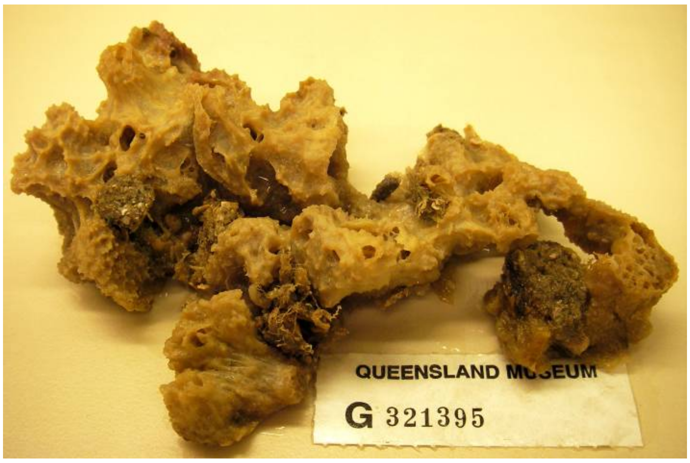

G321395

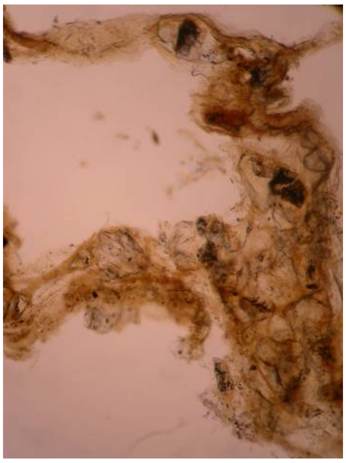

G321395

Psammocinia sp106

Material examined
QMG320893: Gulf of Carpentaria/NT/Australia, 15° 20' 2.22" South, 140° 19' 49.98" East, 28m, 2003, Bartlett, C and Cook, S "Southern Surveyor" 2380403 CSIRO "Effects of Trawling", Trawl.

Description
Shape Massive sponge, slightly flattened to give a thick fan like appearance.
Colour Beige in ethanol, turning the ethanol a murky yellow colour.
Oscules Oscules are situated apically along the upper margin. The are also found raised from the wall of the sponge on small mounds raised to a height of approximately 5mm.
Texture and Surface characteristics Easily compressible, this sponge returns to its original shape immediately. The surface is highly conulose, with primary fibres protruding through the surface regularly, giving a spiky impression. These fibres are a darkened yellow/orange colour and can also be seen in cross section in contrast to the beige choanosome.
Ectosome A loose crust of spicules forms a continuous ectosomal layer.
Choanosome The choanosome is obscured by the very large sand grains which are coring the fibres. The mesohyl is consistent and contains a large amount of foreign material.

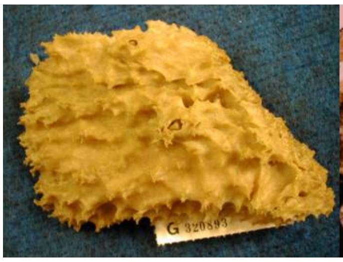
G320893

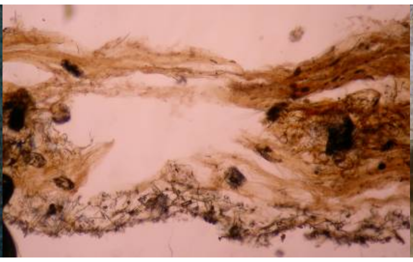
G320893

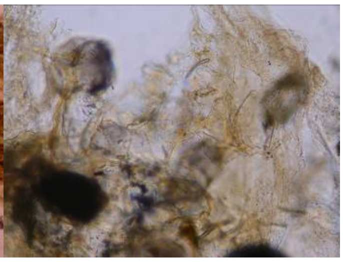
G320893

Psammocinia sp123

Material examined
QMG317230: North Stradbroke Island/QLD/Australia, 27° 28' 6" South, 153° 24' 4" East, 2000, Hooper, J N A, Cook, S D, Kennedy, J A, List-Armitage, S, Edson, D and Woerheide, G, Hand Collection, walking.

Description
Shape Massive, amorphous mass incorporating large bivalves.
Colour Brown exteriore, orange interior.
Oscules Not apparent
Texture and Surface characteristics Flaccid, compressible and dense sponge with shallow conules given an uneven surface appearance.
Ectosome Sand crust composed of very large sand grains.
Choanosome Numerous filamentous algae present within the choanosome, obscuring the skeletal structure.

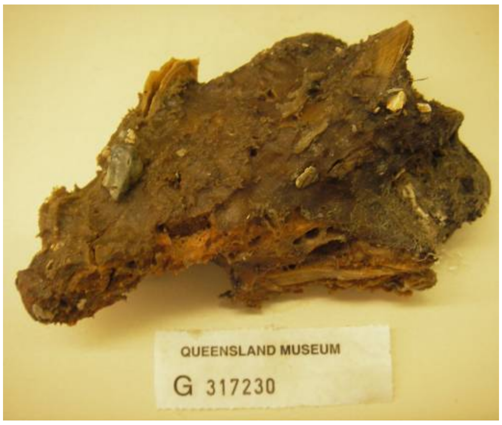

G317230

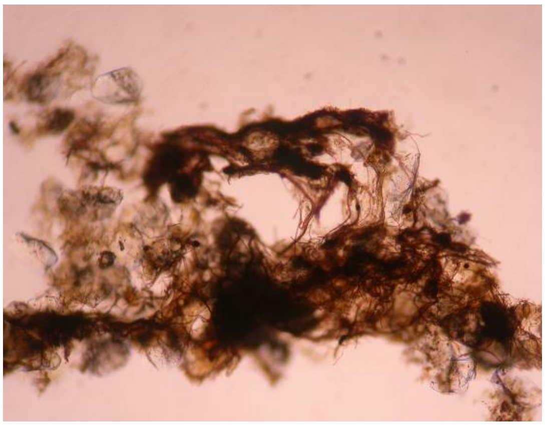

G317230

Psammocinia sp394

Material examined
QMG306028:Cape Jaubert/WA/Australia, 19° 54' 9" South, 117° 12' 1" East, 58m, 1995, Cook, S D on CSIRO RV Southern Surveyor, Trawl. QMG313549: South of Groote Eylandt/NT/Australia, 14° 27' 10.8" South, 136° 14' 16.82" East, 22.5m, 1997, Cook, S D on CSIRO RV Southern Surveyor, Dredge. QMG320901: Gulf of Carpentaria/NT/Australia, 15° 20' 02" North, 140° 19' 50" East, 28m, 2003, Bartlett, C. and Cook, S. "Southern Surveyor" 2380403 CSIRO "Effects of Trawling", Trawl.

Description
Shape Massive subcylindrical barrels.
Colour Grey to light grey or brownish on deck, white to brown in ethanol.
Oscules Large oscules >5mm, scattered over upper surface in between conules, and on the apex of the sponge situated in a terminal depression.
Texture and Surface characteristics Tough, rubbery and difficult to cut. Prominently conulose, with spiky conules up to 2 cm high and occasionally forming longitudinal ridges. Conules terminate in thin, microconulose projections.
Ectosome Contains a thick layer of foreign debris in some parts but debris absent in other parts of the surface.
Choanosome Primary fibres cored with foreign debris, particularly at fibre nodes, and smaller connecting fibres clear of debris. Mesohyl collagen fairly dense but collagenous fibrils very light, not dense, thin, small basal swelling, and mostly visible around the aquiferous canals. Large sand grains and coral detritus also scattered in mesohyl.

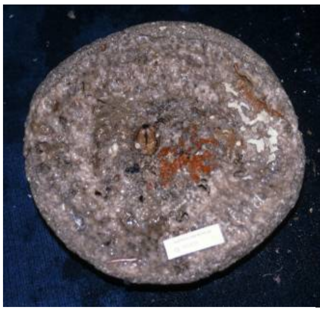
G306028

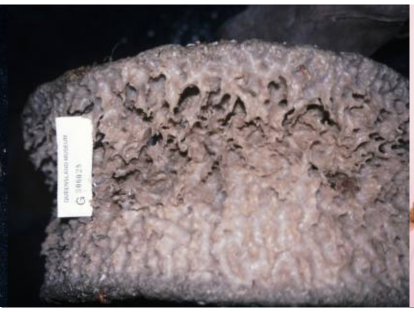
G306028

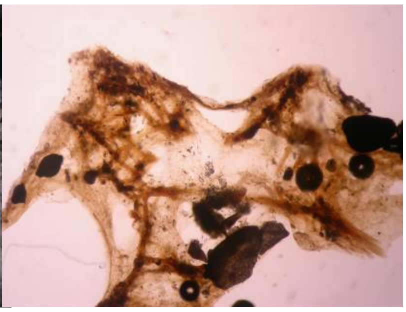
G313549

Psammocinia sp704

Material examined
QMG317738: Gulf of Carpentaria, 21° 30' 00'' South, 152° 26' 06'' East, 30m, , 2001, Hooper, J N A, Cook, S D Kennedy, J A, Woerheide, G, Edson, D, SCUBA.

Description
Shape Bulbous sponges which have a very similar gross morphology to Psammocinia bulbosa.
Colour White externally with yellow, amost pulpy interior.
Oscules Large thin fistules similar to *I.bulbosa*, but partially closed terminally, not open as in *P.bulbosa*. They are also darkened and slightly depressed.
Texture and Surface characteristics Firm and slightly compressible. Uniformly lumpy surface, not conulose.
Ectosome Very thick ectosomal crust of sand.
Choanosome Highly abundant sand, mostly within large fibres. High density of collagenous filaments.

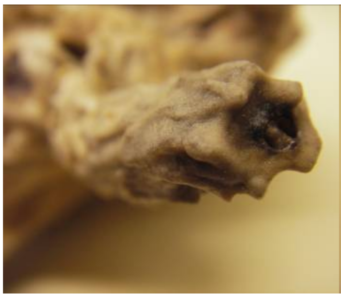

G317738

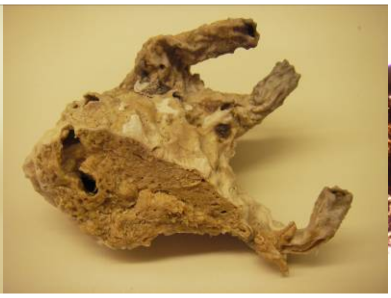

G317738

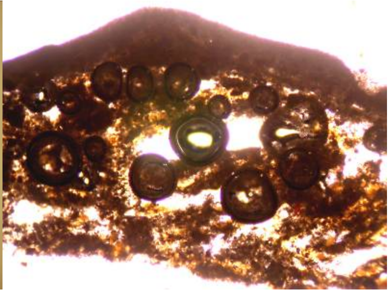

G317738

Psammocinia sp1254

Material examined
QMG316895: Torres Strait/QLD/Australia, 10° 27' 0" South, 142° 55' 12" East, 19m, 2004, CSIRO Torres Strait Map_GM_01_2004 RV Gwendoline May, Trawl.

Description
Shape Massive to vasiform or irregularly digitate.
Colour Black to grey colour in situ, turning to grey / brown on deck and preserved. Turns ethanol a deep orange colour.
Oscules On apex of mass.
Texture and Surface characteristics Prominent conules, interconnected, in some regions, ranging to smooth in others.
Ectosome Arenaceous ectosome with small sand grains and numerous foreign spicule inclusions.
Choanosome Widely spaced fibres almost fully cored with detritus. Abundant collagenous filaments.

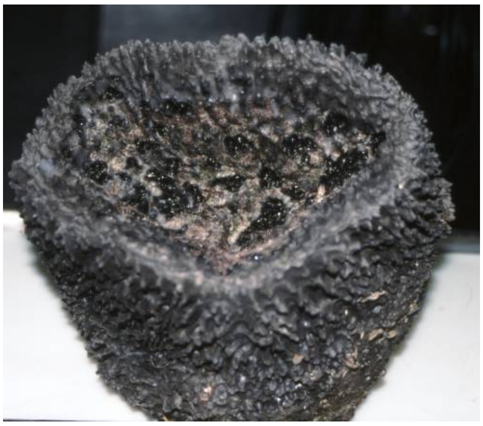

G300777

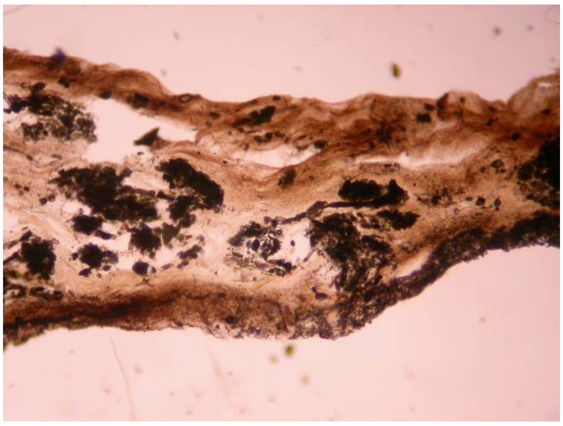

G316895

Psammocinia sp1255

Material examined
QMG316894: Torres Strait/QLD/Australia, 10° 27' 0" South, 142° 55' 12" East, 19m, 2004, CSIRO Torres Strait Map_GM_01_2004 RV Gwendoline May, Trawl.

Description
Shape Massive, subspherical, highly conulose.
Colour White or grey-white.
Oscules Large, in sieve-plate on apical depression. Regularly darkened around the oscular areas.
Texture and Surface characteristics Tough, as "old boots". Highly conulose macroscopically, with fine tracery of sand microscopically with thin arenaceous layer.
Ectosome Regular crust of sand present.
Choanosome Widely spaced primary fibres fully cored with detritus, smaller secondaries uncored, heavy mesohyl with abundant collagen filaments [predominantly with spicule fragments rather than sand coring fibres].

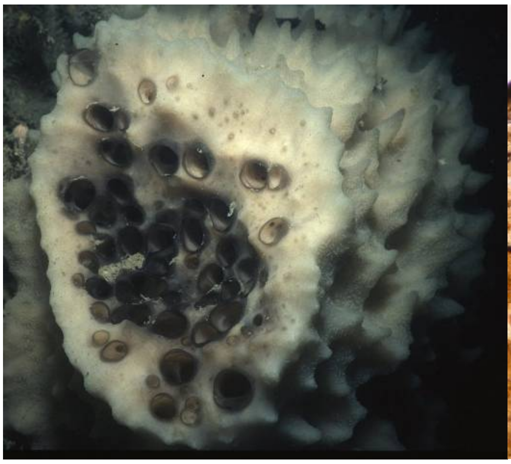

G303422

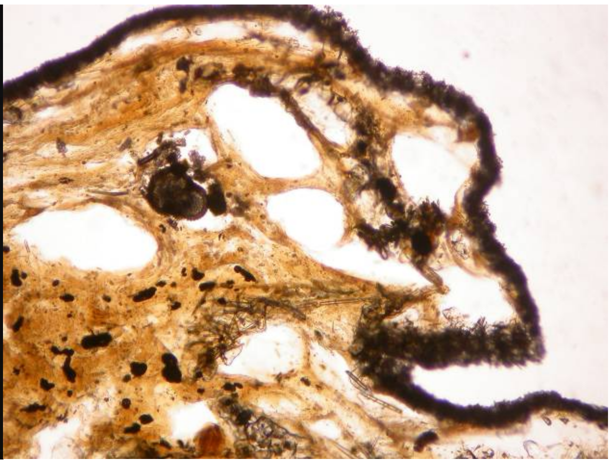

Z1288

Psammocinia sp1513

Material examined
QMG304201: Lizard Island/QLD/Australia, 14° 39' 0" South, 145° 27' 0" East, 18m, 1994, Hooper, J N A, Hobbs, L J, Kennedy, J and Cook, S D, SCUBA.

Description
Shape Flattened, irregular, digitate projections rise from a common base and are often connected to each other. These are erect in situ, but shape is largely lost when preserved in ethanol.
Colour Light brown underwater, with darkened areas around the oscules a smooth, consistent brown colour. Pigmentation is more pronounced at the tips of the conules also. Black on deck and beige in ethanol.
Oscules Terminal to digits, oscules are slightly raised. This becomes less apparent in ethanol and in some areas can simply look as though they are randomly placed across the surface.
Texture and Surface characteristics Soft, flaccid sponge which is compressible. When compressed, it does not regain its original shape, it simply remains flattened. Microscopically areolate pattern giving a cobweb like appearance between the conules which are spread regularly over the surface to produce a gooseflesh appearance. Ectosome is visibly distinct from the choanosome.
Ectosome Thin crust of sand forms a rounded reticulation at the surface.
Choanosome Primary fibres are consistently cored with sand grains. Remainder of the choanosome is relatively free of any foreign material. The mesohyl is pigmented a consistently brown colour.

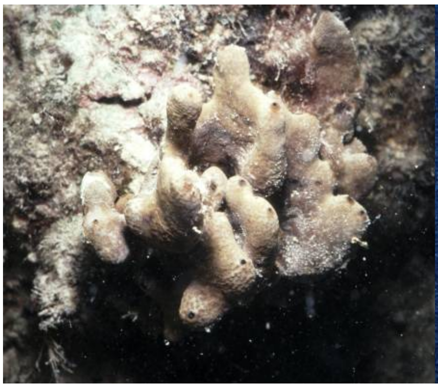

G304116

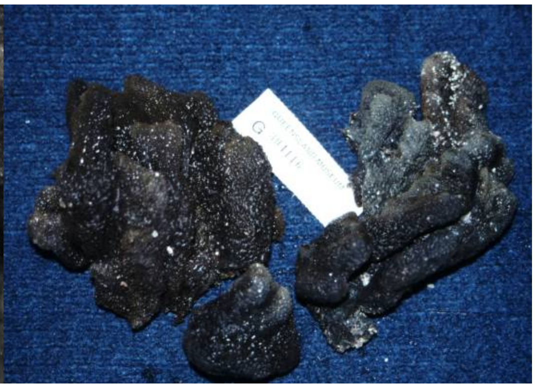

G304116

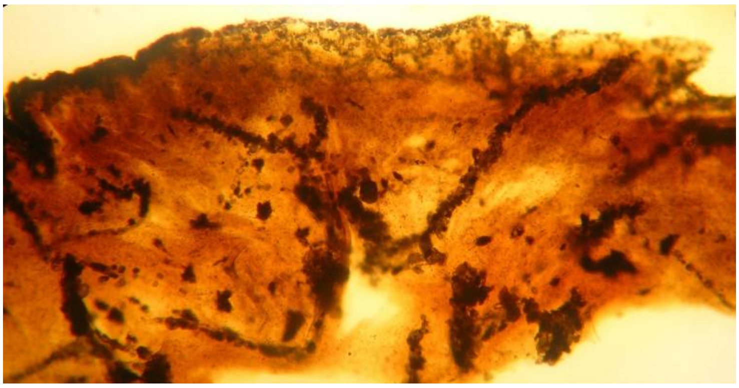

G304116

Psammocinia sp1909

Material examined

QMG315620: Bare Island/ NSW/Australia, 33° 59' 38" South, 151° 13' 60" East, 7m, 1999, Taylor, M University of New South Wales, SCUBA. QMG315621: Bare Island/ NSW/Australia, 33° 59' 38" South, 151° 13' 60" East, 7m, 1999, Taylor, M University of New South Wales, SCUBA.

Description

Shape Thick, erect, lamellate ridge.
Colour Light beige alive, dark brown on deck and grey in ethanol. Internally, the tissue takes on a slightly orange coloration.
Oscules Apex of ridge appears as a continuous sieve-plate containing many small oscules.
Texture and Surface characteristics Tough, compressible, difficult to tear. Opaque, membranous surface which is uneven with prominent irregular conules. Cavernous internally, which can be easily seen when cut.
Ectosome Membranous, with thin crust of detritus arranged in small circular meshes.
Choanosome Irregular , widely spaced fasciculations of primary and secondary fibres mainly obscured by the abundant knobbed collagen filaments in the mesohyl. Primary fibres are fully charged with large sand detritus; secondary fibres are clear. Laminations and pith in fibres are not obvious due to their being obscured by collagen filaments.

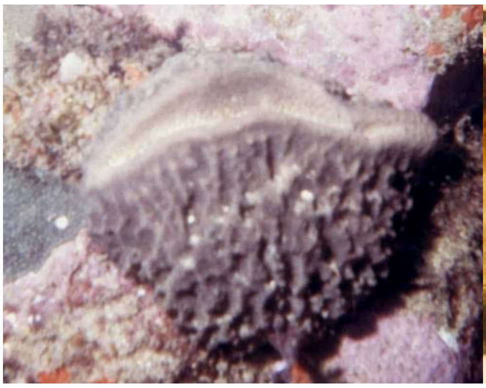

G307109

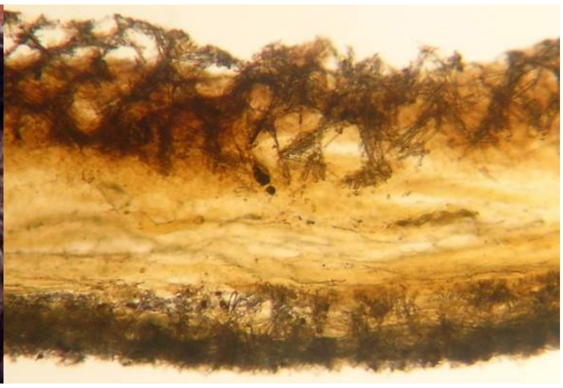

G307054

Psammocinia sp1944

Material examined
QMG319395: Cape Grafton/QLD/Australia, 16° 48' 33.66" South, 146° 12' 46.79" East, 32m, 2002, Hooper, J N A, Schlacher, M, Woerheide, G and Carroll, A et al, SCUBA.

Description
Shape Massive sponge tapering at the basal attachment.
Colour Brown in ethanol, lighter internally. Turns the ethanol an orange colour. A thick layer of white matter has been excreted from the sponge and is coagulated at the bottom of the jar.
Oscules Tiny oscules located apically are slightly raised. The surrounding ectosome is darkened to an almost black colour.
Texture and Surface characteristics Extremely firm and dense, but compressible. Ethanol squirts out of the apical oscules when compressed. Regularly conulose, medium sized conules. Tips of the conules are elongated, thick and pointed.
Ectosome Thick crust of foreign spicules is not very dense, but continuous and homogenous.
Choanosome Light mesohyl present consistenly throughout the choanosome, with light but homogenous inclusion of broken foreign spicules. Large, uncored fibres run sparsely through the choanosome, with foreign material concentrated at the extremities of the fibres. This gives the fibres an appearance of a circular arrangement of spicules, with a hollow centre, when in cross section.

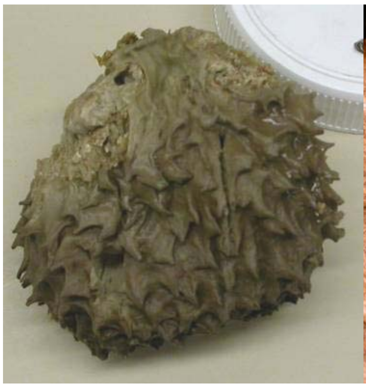

G319395

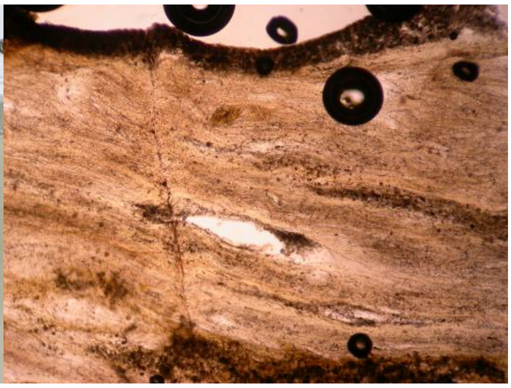

G319395

Psammocinia sp2188

Material examined

QMG307522: North side Polmaise Reef/QLD/Australia, 23° 33' 3" South, 151° 39' 9" East, 12m, 1996, Hooper, J N A, Cook, S D, Kennedy, J A and Tomkins, P A, SCUBA. QMG307533: North side Polmaise Reef/QLD/Australia, 23° 33' 3" South, 151° 39' 9" East, 12m, 1996, Hooper, J N A, Cook, S D, Kennedy, J A and Tomkins, P A, SCUBA.

Description

Shape Thickly encrusting, with short fistules.
Colour Black with beige base alive, on deck, and in ethanol.
Oscules Singular, moderately small oscules which are located apically, on short fistules.
Texture and Surface characteristics Tough, compressible and difficult to tear. This species is fleshy, collagenous, heavily pigmented and uneven, with conulose surface.
Ectosome Membranous, arenaceous, with sand grains and foreign spicule fragments incorporated into peripheral fibres. Membrane has abundant pigment granules incorporated into the collagen.
Choanosome Irregular reticulation of primary fibres comprise most of the choanosomal fibre skeleton, but some secondary fibres also occur. Primary fibres are fully cored by sand and foreign spicule detritus and are commonly fasciculate. Secondary fibres are simple and lack coring material. All fibres are lamellate and contain a diffuse pith. Knobbed collagen filaments are abundant throughout the choanosome, and moderately well developed. Mesohyl collagen is light, homogenous and contains small pigment granules.

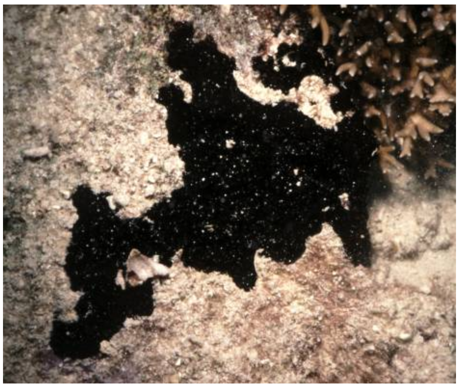

G307522

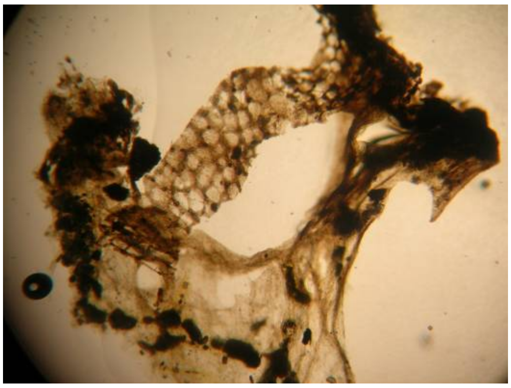

G307522

Psammocinia sp2268

Material examined
QMG307901: Wooded Islet/QLD/Australia, 16° 23' 4" South, 145° 33' 8" East, 1997, Hooper, J N A, Cook, S D, Kennedy, J A, Tomkins, P A and List-Armitage, S, SCUBA.

Description
Shape Lobate stoloniferous, spreading over substrate, with erect, blind tapering fistules. Looks superficially like *Dysidea herbacea*.
Colour Live colouration unknown; dark olive-green on deck; light orange-grey in ethanol.
Oscules Few, small oscules located in between fistules on the surface.
Texture and Surface characteristics Firm, compressible, slightly harsh sponge with an opaque, microconulose surface.
Ectosome Membranous ectosome with an even scattering of fine sand-grain detritus. Surface is regularly pushed up into microconules by ascending choanosomal fibres.
Choanosome Irregular reticulation of fibres fully cored by relatively large sand-grain detritus. Fibres are not readily distinguishable into primary or secondary fibre categories. Knobbed collagen fibrils are abundant in the choanosome, with some pigmented slightly darker than others making them somewhat more obvious. Mesohyl collagen is moderately light and homogenous, and lacks foreign inclusions.

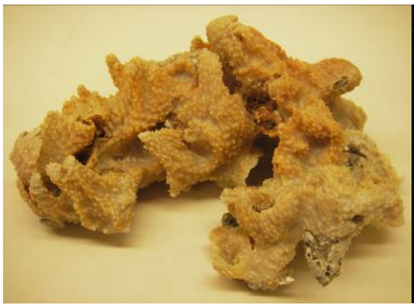

QMG307901

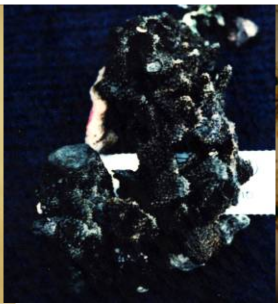

QMG307901

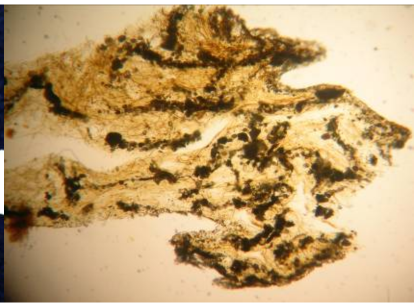

QMG307901

Psammocinia sp2400

Material examined
QMG313546: South of Groote Eylandt/NT/Australia, 14° 27' 10.8" South, 136° 14' 16.82" East, 22.5m, 1997, Cook, S D on CSIRO RV Southern Surveyor, Dredge.

Description
Shape Numerous, erect, fused, tapering digits attached to a common, stalked base.
Colour Beige on deck and in ethanol.
Oscules Not visible.
Texture and Surface characteristics Tough, compressible, difficult to tear. Opaque, membranous, optically smooth surface. Uneven, conulose, prominently sculptured with ridges running between surface conules.
Ectosome Membranous, with a fine scattering of sand and spicule detritus amongst knobbed collagen filaments from the choanosome that are embedded in the surface membrane.
Choanosome Irregular reticulation of primary and secondary fibres that contain a scattering of larger sand detritus that may occasionally expand the fibres. All fibres are lightly pithed and laminated. Mesohyl contains abundant, large, knobbed collagen filaments and generally lacks foreign inclusions.

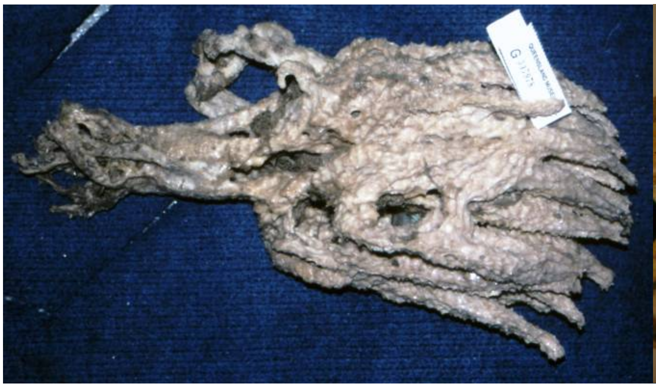

G307978

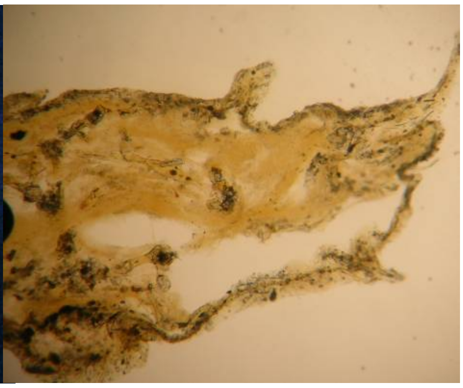

G307978

Psammocinia sp2667

Material examined
QMG315068: Sunshine Coast/QLD/Australia, 26° 39' 11.4" South, 153° 10' 59.78" East, 19m, 1998, Hooper, J N A, Cook, S D, Kennedy, J A and Woerheide, G, SCUBA.

Description
Shape Encrusting sponge forming bulbous projections which appear to partially collapse in ethanol.
Colour Black in situ, turning an even cream colour in ethanol.
Oscules Oscules are apical to bulbous sections, which is highly obvious in situ but less pronounced in ethanol. Oscule lips are slightly raised.
Texture and Surface characteristics Conulose surface with tips of the conules forming sharp points. Soft and easily compressible and subsequently returns to shape quickly. The conules are connected by a thin membranous layer which can be opaque.
Ectosome Darkened but not highly sandy crust.
Choanosome Thin but highly consistent coring of primary fibres. The coring does not obstruct or affect the external structure of the fibres, but is kept wholly within the fibre. This leads into coring of secondary fibres, which is less consistent. Primary and secondary fibres from a wide meshed reticulation with a dense network of collagenous filaments and consistent constituting the majority of the choanosome.

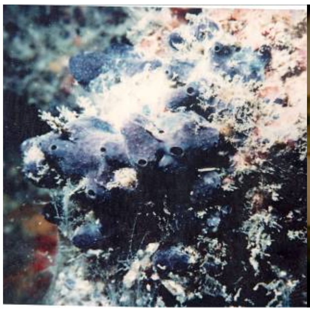
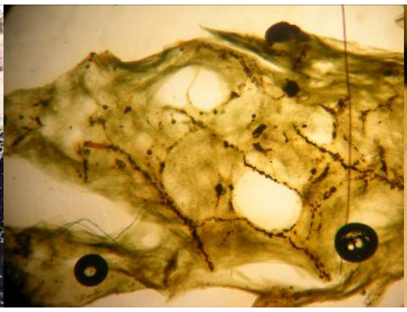
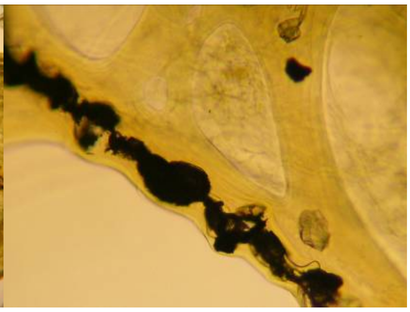

QMG315068 QMG315068 QMG315068

Psammocinia sp2767

Material examined
QMG314618: Gold Coast/QLD/Australia, 27° 57' 1" South, 153° 26' 58.78" East, 26m, 1999, Cook, S D, Kennedy, J A, Adams, C L and Woerheide, G, SCUBA. QMG315081: Sunshine Coast/QLD/Australia, 26° 39' 11.4" South, 153° 10' 59.78" East, 19m, 1998, Hooper, J N A, Cook, S D, Kennedy, J A and Woerheide, G, SCUBA. QMG316873: Torres Strait/QLD/Australia, 10° 15' 0" South, 142° 55' 48" East, 22.1m, 2004, CSIRO Torres Strait Map_GM_01_2004 RV Gwendoline May, Trawl. QMG317710: Swain Reefs/QLD/Australia, 21° 29' 53.46" South, 152° 24' 52.05" East, 15m, 2001, Hooper, J N A, Cook, S D, Kennedy, J A, Edson, D and Woerheide, G, SCUBA. QMG317993: NE of Yeppoon/QLD/Australia, 22° 58' 26.4" South, 151° 10' 59.4" East, 35m, Queensland Department of Primary Industries Fisheries, Deception Bay (QDPI), Trawl. QMG319419: East of Gladstone/QLD/Australia, 23° 51' 35.4" South, 151° 41' 23.4" East, 30m, Queensland Department of Primary Industries Fisheries, Deception Bay (QDPI), Trawl. QMG319474: NE of Burnett Heads/QLD/Australia, 24° 32' 37.2" South, 152° 41' 28.79" East, 28m, Queensland Department of Primary Industries Fisheries, Deception Bay (QDPI), Trawl. QMG320235: QDPI Scallop Survey/QLD/Australia, 23° 27' 4.42" South, 151° 28' 22.06" East, 30m, 2002, Queensland Department of Primary Industries Fisheries, Deception Bay (QDPI), Trawl. QMG320908: Gulf of Carpentaria/QLD/Australia, 15° 20' 2.22" South, 140° 19' 49.98" East, 28m, 2003, Bartlett, C and Cook, S "Southern Surveyor" 2380403 CSIRO "Effects of Trawling", Trawl. QMG321037: Torres Strait/QLD/Australia, 9° 50' 24" South, 143° 14' 24" East, 25.4m, 2004, CSIRO Torres Strait Map_GM_01_2004 RV Gwendoline May, Trawl.

Description
Shape Subspherical mound to barrel shaped with large conules.
Colour Dark grey ectosome and beige choanosome in situ, same in alcohol. Mostly darker towards apex (dark grey/black) to lighter at base, but some specimens quite white/beige all over.
Oscules Scattered oscules, with some aggregated oscular areas. Apical depressions with darkened oscules at base of depression.
Texture and Surface characteristics Firm yet compressible, dense and soggy. The surface is membranous, hispid and translucent.
Ectosome Crust of medium to large sized sand grains.
Choanosome Dense with filaments, some anastomosing fibres, but obscured by filaments.

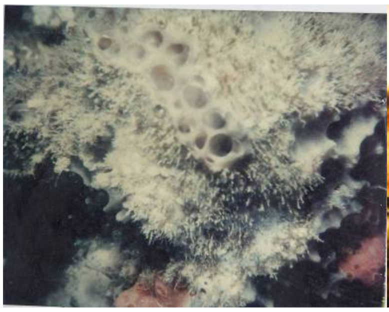
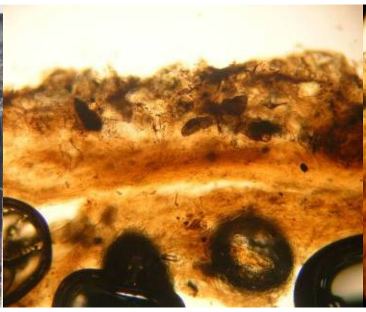
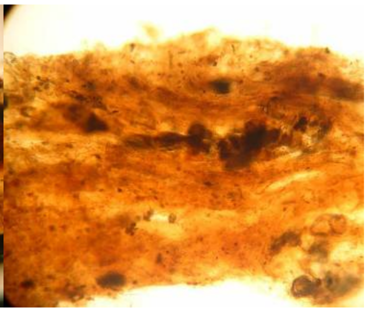

QMG314618 QMG314618 QMG314618

Psammocinia sp2901

Material examined
QMG315823: Sunshine Coast/QLD/Australia, 26° 22' 32" South, 153° 7' 33" East, 29m, 1999, Cook, S D, Kennedy, J A, List-Armitage, S E, Adams, C L and Woerheide, G, SCUBA.
QMG320131: Keppel Islands/QLD/Australia, 23° 7' 5" South, 150° 59' 27" East, 17.4m, 2002, Hooper, J N A, Cook, S D, List-Armitage, S, Richer de Forges, M, Crowther, A and Ireland, H, SCUBA.

Description
Shape Massive
Colour Dark grey on deck and in ethanol.
Oscules Few large oscules, flush with the surface.
Texture and Surface characteristics Firm yet compressible. The surface is heavily encrusted and uneven but regularly covered in microconules.
Ectosome Tightly woven fibrils with layer of sand tangential to the surface.
Choanosome Large, fasciculating fibres cored with detritus. Many fibrils tightly woven with abundant detritus

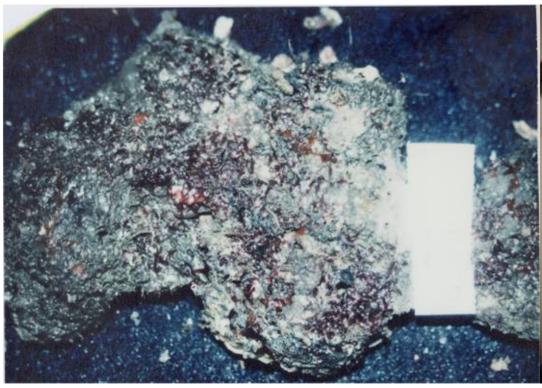

G315823

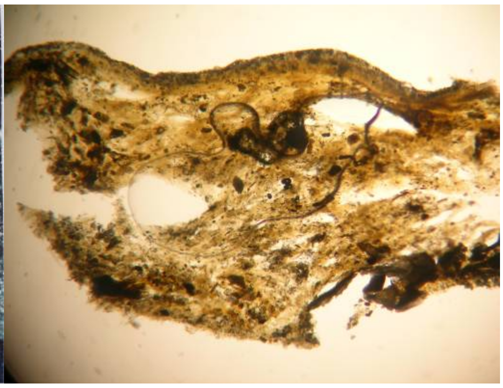

G315823

Psammocinia sp2936

Material examined
QMG318393: Pompey Reefs/QLD/Australia, 21° 12' 45" South, 151° 11' 43" East, 21.8m, 2000, Cook, S D, Kennedy, J A, Woerheide, G and Delaney, W, SCUBA.

Description
Shape Massive.
Colour Dark tan/brown alive. Light brown in preservative, turning the ethanol a fluorescent yellow colour.
Oscules Small, regularly distributed over the surface, with slightly raised, membranous lip.
Texture and Surface characteristics Firm, compressible sponge which is difficult to tear. The surface is slightly opaque and prominently conulose. In preservative, some of the oscules are coloured a very dark brown colour, giving a spotted appearance.
Ectosome Very thick crust of sand at the cortex.
Choanosome Few fibres present. The choanosome is comprised mainly of fine, knobbed collagen filaments with veins of fine sand detritus and scattered sand throughout.

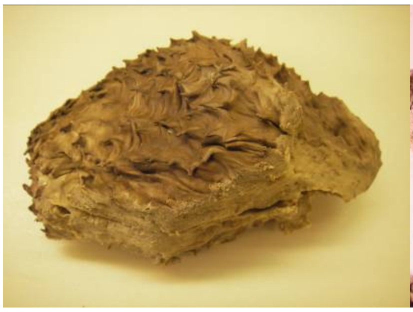
G318393

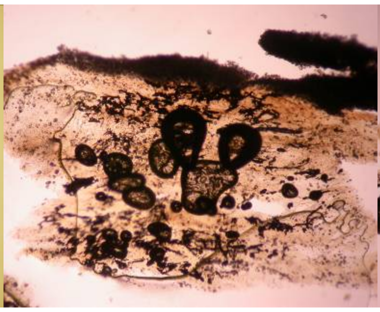
G318393

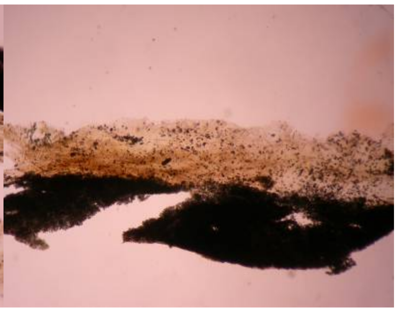
G318393

Psammocinia sp3077

Material examined
QMG319523: NE of Gladstone/QLD/Australia, 23° 36' 57" South, 151° 38' 45.59" East, 36m, Queensland Department of Primary Industries Fisheries, Deception Bay (QDPI), Trawl.

Description
Shape Massive sponge.
Colour Red on deck, turning to cream in ethanol. Large primary and secondary fibres can be seen and are a contrasting orange/brown colour.
Oscules Conules are connected by a very thin, membranous layer. Oscules can be seen forming an almost perfectly rounded shape within these areas. Occasionally, the membranous layer is absent and the canal is visible, running deeply within the body of the sponge.
Texture and Surface characteristics Extremely cavernous sponge with a thin membranous surface layer which is regularly absent. In these instances, the membranous webbing runs perpendicular to the surface and thin tendrils of membrane connect the conules like bridges.
Ectosome Sand filled crust forms a thin surface layer.
Choanosome Large primary and secondary fibres form a wide meshed, regular reticulation which is heavily cored with very large sand grains. The mesohyl is filled with granules.

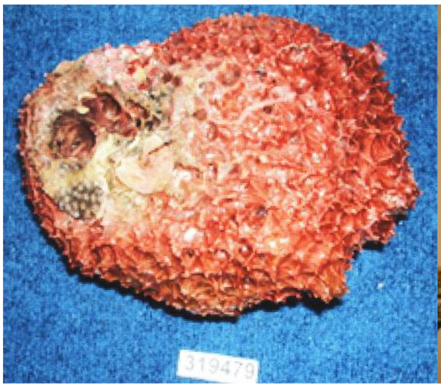

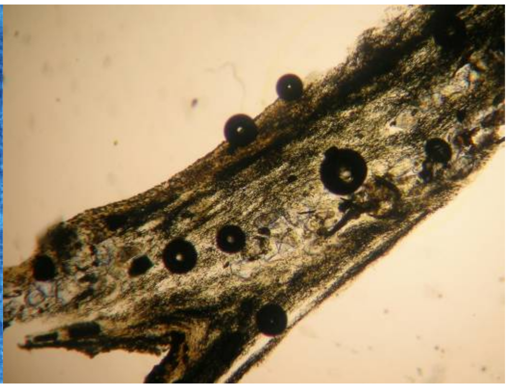

G319479 G319479

Psammocinia sp3176

Material examined

QMG315808: Star Reef/QLD/Australia, 26° 22' 12'' South, 153° 07' 30'' East, 29m, 1999, Cook, S. D., Kennedy, J. A., List-Armitage, S. E., Adams, C. L. and Woerheide, G., SCUBA.

Description

Shape Very similar to *P. halmiformis*, however has a pedunculate growth form rather than irregular cup shape.
Colour Almost identical to *P. halmiformis*, brown on one side, slightly more beige on the other. Internally white.
Oscules One side completely covered in small oscules, flush with the surface. Very regular distribution.
Texture and Surface characteristics Firm, dense, not very compressible, but slightly flexible. One side smooth to touch, but grossly undulating. Outside surface is also undulating and microconulose. Where cut, the beige choanosome is visible, with thick yellow fibres very prominent.
Ectosome Very uniform, thick, sand ectosome.
Choanosome Very different to *P. halmiformis*, which has smaller sand grains, coring only primary fibres and secondary at intersections. This species has very large sand grains throughout whole slide, no good localisation in fibres.

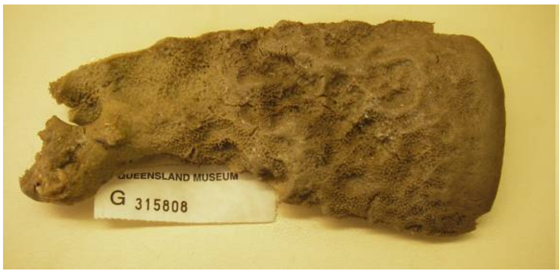

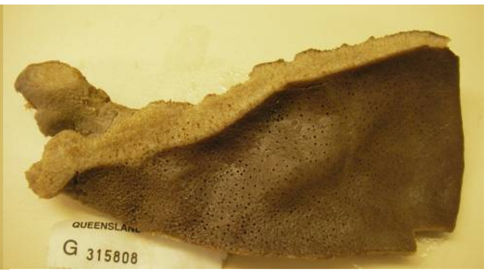

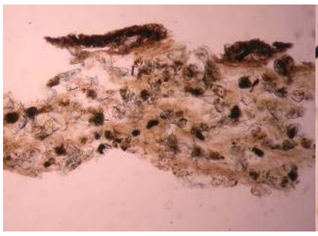

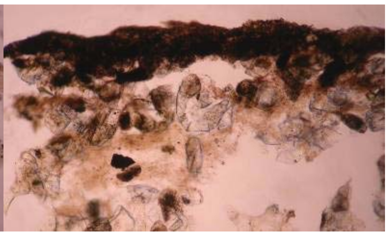

G315808 G315808

Psammocinia sp3983

Material examined

QMG321264: Southern Gold Coast/QLD/Australia, 28° 6' 27.3" South, 153° 28' 37.8" East, 12.5m, 2004, Crowther, A, Ekins, M, Carini, G and Sutcliffe, P, SCUBA. QMG321268: Southern Gold Coast/QLD/Australia, 28° 6' 27.3" South, 153° 28' 37.8" East, 12.5m, 2004, Crowther, A, Ekins, M, Carini, G and Sutcliffe, P, SCUBA. QMG321391: Palm Reef/QLD/Australia, 28° 6' 33.12" South, 153° 28' 30.96" East, 10m - 24m, 2004, Crowther, A, Ekins, M, Carini, G and Sutcliffe, P, SCUBA. QMG321399: Palm Reef/QLD/Australia, 28° 6' 33.12" South, 153° 28' 30.96" East, 10m - 24m, 2004, Crowther, A, Ekins, M, Carini, G and Sutcliffe, P, SCUBA.

Description

Shape Massive sponge rising to crested ridges.
Colour Dark grey on deck and in ethanol.
Oscules Arranged apically along the ridges.
Texture and Surface characteristics Rubbery and extremely difficult to tear. Conulose surface.
Ectosome Layer of darker pigmented ectosome with some sand grains present.
Choanosome Large, uncored, clear, laminated fibres. Distinct filaments forming whirls of wool

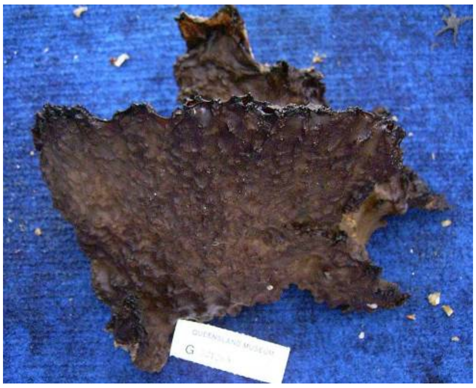

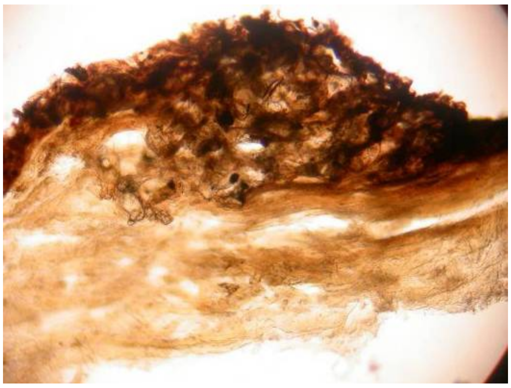

G321268 G321268
